# DNA Polymerase θ Drives Colorectal Cancer Progression through Wnt/β-Catenin Activation and Shows Potential Association with Immunosuppressive Microenvironment Remodeling

**DOI:** 10.32604/or.2026.080204

**Published:** 2026-09-14

**Authors:** Li Li, Jianhua Wang, Feilong Zhou, Lingjun Geng, Kongwang Hu

**Affiliations:** 1 Department of General Surgery, The Fifth Affiliated Hospital of Anhui Medical University, Fuyang, China; 2 Department of General Surgery, The Second Affiliated Hospital of Nanjing Medical University, Nanjing, China

**Keywords:** POLQ (DNA polymerase theta), colorectal cancer, Wnt/β-catenin signaling, epithelial-mesenchymal transition (EMT), tumor microenvironment

## Abstract

**Background:** Colorectal cancer (CRC) is a leading cause of cancer-related mortality worldwide, with limited treatment options for advanced-stage patients. DNA polymerase theta (POLQ) is overexpressed in various cancers, but its role and underlying mechanisms in CRC remain not fully elucidated. This study aimed to investigate the expression, prognostic value, biological functions, and molecular mechanisms of POLQ in CRC. **Methods:** POLQ expression was analyzed using public databases and 55 paired clinical samples. Lentivirus-mediated knockdown and overexpression were employed to assess CRC cell proliferation, migration, and invasion. Single-cell transcriptomics and CellChat analysis were used to explore the tumor microenvironment (TME). Western blotting, immunofluorescence, and dual-luciferase reporter assays were performed to verify the Wnt/β-catenin pathway and epithelial-mesenchymal transition (EMT). **Results:** POLQ was significantly overexpressed in CRC tissues and cell lines, and high POLQ expression was associated with neural invasion, vascular tumor thrombus, lymph node metastasis, advanced TNM stage, and poor prognosis. Single-cell analysis revealed that POLQ was specifically enriched in malignant epithelial cells, which acted as communication hubs with prominent Wnt signaling. Functional experiments demonstrated that POLQ activated the Wnt/β-catenin pathway, induced EMT, promoted β-catenin nuclear translocation and TCF/LEF transcriptional activity, and enhanced CRC cell proliferation, migration, and invasion. The Wnt inhibitor XAV939 reversed the POLQ overexpression-induced malignant phenotype, while the Wnt activator SKL2001 partially rescued the knockdown phenotype. Additionally, single-cell transcriptomics and CellChat analysis generated a computational prediction that POLQ-high cells may be involved in remodeling of the immunosuppressive microenvironment; however, this inference requires direct experimental validation through immune cell profiling in future studies. **Conclusions:** POLQ promotes CRC progression by activating the Wnt/β-catenin-EMT axis. Bioinformatic inference suggests a potential association with an immunosuppressive microenvironment, but this finding remains correlative and requires experimental confirmation. These findings suggest that POLQ is a potential prognostic biomarker and therapeutic target, though further validation in prospective, multicenter cohorts is warranted.

## 1 Introduction

Colorectal cancer (CRC) is a highly prevalent gastrointestinal malignancy globally, ranking as the third most commonly diagnosed cancer and the second leading cause of cancer-related deaths [1]. Projections from the GLOBOCAN database indicate that the annual number of new CRC cases will rise to 3.2 million by 2040, accompanied by an estimated 1.6 million deaths globally [2]. This will place an even heavier burden on society and the public health system. Despite significant advancements in CRC screening and early diagnosis, approximately 23% of patients still present with metastatic disease at initial diagnosis. Consequently, the five-year overall survival (OS) rate for metastatic CRC (mCRC) remains below 15% [3]. Current clinical treatment is still based on surgical resection combined with radiotherapy. Targeted therapies directed against the epidermal growth factor receptor (EGFR) and vascular endothelial growth factor receptor (VEGFR) signaling pathways have significantly improved overall survival in specific patient subsets [3]. However, their efficacy is limited and they can only improve the prognosis of specific patient groups. Recently, immune checkpoint inhibitors (ICIs), particularly PD-1/PD-L1 blockers, have revolutionized the treatment landscape for various malignancies, including CRC [4]. However, ICIs only benefit 15% of CRC patients characterized by defective mismatch repair/high microsatellite instability (dMMR/MSI-H) [5]. Therefore, exploring the molecular mechanisms of CRC development in depth and identifying new therapeutic targets and prognostic biomarkers are important for achieving personalized, precise treatment and improving patient survival.

POLQ is an evolutionarily conserved protein encoded by the POLQ gene in the mammalian genome. POLQ is a multi-domain protein composed of an N-terminal helicase-like domain, a non-conserved central domain, and a C-terminal polymerase domain [6]. The function of POLQ was first revealed in studies of Drosophila, where mutations in its immediate homologue, MUS308, result in loss of viability when exposed to DNA breakage inducers such as cross-linking agents or radiation [7]. Due to its low fidelity and ability to perform nucleotide insertions and extensions across DNA lesions such as abasic sites and thymine glycol, it plays a key role in the defense against double-strand breaks (DSBs) induced by various factors, including ionizing radiation, replication fork stalling, and CRISPR-Cas9, thereby maintaining genomic stability [8]. Studies have demonstrated that POLQ mediates a microhomology-mediated and error-prone DSB repair pathway, termed theta polymerase-mediated end joining (TMEJ) [9,10]. Unlike classical homologous recombination repair (HR) or non-homologous end-joining (NHEJ) pathways, TMEJ generates non-homologous junctions with a considerably higher template insertion frequency [11,12]. These junctions provide microhomologies that can bridge the two DSB ends and compensate for deficiencies in high-fidelity HR repair under conditions of DNA damage.

In normal cells, POLQ is generally expressed at very low or undetectable levels. However, it is overexpressed in various human cancers (including ovarian, breast, lung, gastric, head and neck, and colorectal cancers), and its high expression is closely associated with a poor clinical prognosis [13]. For instance, POLQ has been demonstrated to promote tumor progression by regulating cell proliferation, apoptosis and migration in hepatocellular carcinoma (HCC). Furthermore, high POLQ expression in tumor tissues has been significantly associated with adverse clinical features, such as multifocality, an increased risk of recurrence, and elevated alanine aminotransferase/gamma-glutamyl transferase (ALT/GGT) levels, suggesting its potential as a prognostic marker [14]. In prostate cancer, POLQ expression is associated with postoperative recurrence and poor prognosis [13]. Of particular significance was the finding that the inhibition of POLQ reversed the resistance of HR-deficient tumors to PARP inhibitors (PARPi) [15], suggesting it as a potential new strategy for the targeted therapy of HR-deficient tumors. However, to date, the expression level of POLQ in CRC, its specific biological functions in CRC cell lines, and the related molecular mechanisms have not been systematically and thoroughly elucidated.

It is worth further exploring whether the oncogenic role of POLQ extends beyond its classical DNA damage repair function to include the regulation of key signaling pathways. Among these, the Wnt/β-catenin signaling pathway is one of the most critical drivers of CRC pathogenesis and progression of CRC. This pathway activates transcription factors such as Snail through the β-catenin/TCF (T-cell factor)/LEF (lymphoid enhancer-binding factor) transcriptional complex, thereby inducing EMT [16,17]. As a key hallmark of cancer progression, EMT enhances the migration and invasion capabilities of tumor cells and serves as a crucial driver of CRC recurrence and metastasis [18,19]. Furthermore, EMT plays a significant role in the clinical diagnosis and prognostic assessment of CRC [20,21]. Notably, a study in breast cancer revealed that the EMT-related transcription factor ZEB1 can directly inhibit POLQ expression, thereby modulating TMEJ repair activity and maintaining genomic stability and integrity [22]. This finding suggests a potential feedback regulatory mechanism between POLQ and EMT. In contrast, this study aims to explore whether POLQ affects the EMT process in colorectal cancer by regulating the Wnt/β-catenin signaling pathway, thereby exerting its tumor-promoting effect. This may reflect tissue-specific functions of POLQ in different cancer types. Whether these regulatory differences stem from distinct tumor microenvironments or signaling pathway contexts in breast versus colorectal cancer warrants further investigation.

In the present study, we aimed to investigate the expression and clinical significance of POLQ in colorectal cancer (CRC), determine its effects on the malignant phenotypes of CRC cells, and test the hypothesis that POLQ promotes EMT and CRC progression through activation of the Wnt/β-catenin signaling pathway. This study also aimed to identify POLQ as a novel biomarker for early CRC diagnosis and prognostic assessment, provide a theoretical basis for its development as a therapeutic target, and advance precision treatment for colorectal cancer.

## 2 Materials and Methods

### 2.1 Single-Cell Transcriptomic Processing and Differential Expression Analysis

This study included the GEO datasets GSE132465 and GSE144735. GSE132465 contains 18 CRC samples (10 primary tumors and 8 matched metastatic lesions), and GSE144735 contains 12 CRC samples (all primary tumors). Using R (v4.2.0, R Foundation for Statistical Computing, Vienna, Austria), the original or processed gene-cell count matrices were analyzed with Seurat (v4.1.1, Satija Lab, New York, NY, USA). Data normalization was performed using the LogNormalize method implemented in Seurat (normalization.method = “LogNormalize”, scale factor = 10,000). Cells exhibiting low transcriptional complexity or poor quality were excluded based on the following criteria: fewer than 1000 detected UMIs or a mitochondrial gene proportion exceeding 15%. After filtering, the retained cell numbers were 15,824 cells for GSE132465 and 11,237 cells for GSE144735. Potential doublets were identified and removed by DoubletFinder (v2.0.3) for each sample (expected doublet rate was set to 5%, and the optimal PC number was determined based on pK estimation). Following normalization and the identification of highly variable genes, batch correction was performed using Harmony (v0.1.0, Immunogenomics Group, Boston, MA, USA) to mitigate inter-sample variability. The top 2000 highly variable genes were selected for principal component analysis (PCA), and the first 30 principal components (PCs) were used to construct a shared nearest neighbor graph (k = 20) and perform unsupervised clustering (resolution parameter set to 0.5). Uniform manifold approximation and projection (UMAP) was used for visualization. Cell type annotation was performed by scoring canonical marker gene sets for major populations, including B cells (CD79A, CD79B), epithelial cells (ALPI, FABP1, APOA1, KRT20), stromal cells (COL1A1, COL1A2, DCN, LUM), cancer cells (EPCAM, KRT8, KRT18, KRT19), myeloid cells (CSF3R, C1QB, CD1C, CLEC9A), and T/NK cells (CD3D, CD3E, KLRD1, KLRC1). Differentially expressed genes (DEGs) among clusters were identified using the FindAllMarkers function with parameters set to min.pct = 0.15 and logfc.threshold = 0.15, retaining only positively enriched genes. Statistical significance was assessed using the Wilcoxon rank-sum test, followed by multiple-testing correction with the Benjamini–Hochberg method. In single-cell analysis, cancer cells are stratified based on the detectable expression of POLQ. Cells with POLQ expression greater than 0 are defined as POLQ-positive (POLQ+) cells, while cells with POLQ expression equal to 0 are defined as POLQ-negative (POLQ−) cells.

### 2.2 Functional Enrichment and Gene Set Enrichment Analysis

Functional annotation of DEGs was performed using the clusterProfiler R package (v4.7.1.2, Bioconductor, Boston, MA, USA). Kyoto Encyclopedia of Genes and Genomes (KEGG) pathway enrichment analysis and gene set enrichment analysis (GSEA) were conducted to identify biologically relevant pathways. The KEGG database version 2023 (release 107.0) was used for enrichment analysis. Adjusted *p*-values were calculated using the Benjamini-Hochberg false discovery rate (FDR) correction method. For KEGG and GO analyses, enriched pathways with adjusted *p*-values < 0.05 were deemed statistically significant. In contrast, for GSEA, pathways exhibiting |NES| > 1, nominal *p* < 0.05, and FDR < 0.25 were considered significantly enriched, adhering to established criteria.

### 2.3 Cell–Cell Communication Analysis

Cell-cell communication within the colorectal cancer TME was analyzed using CellChat (version 1.6.1; Suoqin Jin, GitHub, San Francisco, CA, USA), implemented in R. The expression matrix was normalized using the LogNormalize method implemented in the Seurat package (NormalizeData function, scale factor = 10,000) prior to CellChat input. The standardized single-cell expression matrix and cell type annotations were used as input. The analysis utilized the built-in human ligand-receptor interaction database (CellChatDB.human, version v1.0.0 included in CellChat 1.6.1). According to the built-in ligand-receptor interaction database and following the standard CellChat workflow, the analysis was conducted. Specifically, the “computeCommunProb” function was employed to infer communication probabilities with 100 permutations, and “computeCommunProbPathway” was used to aggregate interactions at the signaling pathway level. The “netAnalysis_signalingRole” function was applied to evaluate outgoing and incoming signaling patterns, and the sender/receiver roles were visualized using “netAnalysis_signalingRole_scatter”. Based on default or preset filtering criteria, cell groups with insufficient cell numbers were excluded. The communication probabilities at the signaling pathway and ligand-receptor pair levels were inferred, and permutation tests were performed to evaluate significance. *p*-values from permutation tests were corrected for multiple comparisons using the Benjamini-Hochberg false discovery rate (FDR) method. The netAnalysis function in CellChat was used to calculate the outgoing and incoming communication patterns. The sender and receiver roles of POLQ+ cancer cells in the communication network were evaluated. Ligand–receptor pairs were reported if they exhibited a statistically significant communication probability (adjusted *p* < 0.05) and ranked within the top 10% of interaction strengths across all pairs. The ligand-receptor pairs presented were derived from statistically significant interactions inferred by CellChat.

### 2.4 Survival Analysis

Survival analyses were conducted using the survival R package (version 3.5-7, Terry M. Therneau, Mayo Clinic, Rochester, MN, USA). The specific survival endpoint was OS, defined as the time from diagnosis to death from any cause; patients alive at the last follow-up were censored. Kaplan–Meier survival curves were generated using the survfit function, and differences between groups were assessed using two-sided log-rank tests. Patients were stratified into high-POLQ and low-POLQ expression groups based on the median expression value of POLQ. Subgroup analyses (e.g., by T stage) were performed using the same log-rank test without additional adjustment for multiple comparisons, as exploratory analyses. Clinical pathological parameters were compared between the high-POLQ and low-POLQ expression groups using chi-square tests or Fisher’s exact test, as appropriate.

### 2.5 Cell Lines and Cell Culture

Human normal intestinal epithelial cell line (NCM460, [CBP60989]) and CRC cell lines (SW480 [CBP60421], HCT116 [CBP60423], HT-29 [SCSP-5032]) were purchased from the Cell Bank of the Chinese Academy of Sciences (Shanghai, China). These cell lines and their derived cell lines were cultured in RPMI-1640 medium (Gibco, Thermo Fisher Scientific, 11875119, Waltham, MA, USA) containing 10% fetal bovine serum (FBS; Gibco, Thermo Fisher Scientific, 10091148, Waltham, MA, USA) and 1% penicillin-streptomycin solution (P/S; Gibco, Thermo Fisher Scientific, 15140122, Waltham, MA, USA). Cells were cultured in a 5% CO_2_ humidified incubator at 37°C. All cell lines were characterized by short tandem repeat (STR) sequencing and tested to confirm the absence of mycoplasma contamination.

### 2.6 Cell Transfection

To establish stable colorectal cancer cell lines with either stable knockdown or overexpression of POLQ, the lentivirus-mediated gene transduction method was used. For POLQ knockdown, three independent short hairpin RNA (shRNA) sequences (sh1, sh2, sh3) were designed and inserted into the lentivirus vector; a non-targeting shRNA (shNC) was also constructed as a negative control. For POLQ overexpression, the full-length POLQ coding sequence was cloned into the lentivirus overexpression vector, and the empty vector (Vector) was used as a control. The above recombinant lentivirus plasmids were co-transfected with the helper packaging plasmid into HEK293T cells using Lipofectamine 3000 transfection reagent (Thermo Fisher Scientific, L3000015, Waltham, MA, USA) according to the manufacturer’s protocol, and lentivirus particles with high titer were produced. Viral titers were estimated by quantitative real-time PCR (qRT-PCR) targeting vector sequences in RNA extracted from the producer HEK293T cells, which quantifies vector-derived transcripts as a surrogate for packaging efficiency. All lentivirus stocks achieved estimated titers ranging from 1 × 10^8^ to 5 × 10^8^ TU/mL. Since this method measures intracellular vector transcripts rather than physical viral particles in the supernatant, titers should be interpreted as relative estimates. For infection, cells were seeded at 50% confluence, and lentivirus was added at a multiplicity of infection (MOI) of 10 in the presence of 8 μg/mL polybrene (Sigma-Aldrich, TR-1003, St. Louis, MO, USA) to enhance transduction efficiency. After 24 h of infection, fresh medium containing an appropriate concentration of puromycin (2 μg/mL) was added for stable selection, which was continued for 14 days until the control group cells were completely eliminated. The stably transfected cell lines obtained through screening were verified for POLQ mRNA and protein expression levels by quantitative real-time PCR (qRT-PCR) and Western blotting to confirm the knockdown or overexpression efficiency. After verification, the two shRNA sequences with the highest knockdown efficiency (sh1, sh2) and POLQ-overexpressing cells exhibiting significant overexpression were selected for subsequent functional experiments. The POLQ knockdown sequences are as follows: sh-1:5′-GCAGGAGAATGCAAGCCTACA-3′; sh-2:5′-GGACAAGTCCTGGAAGGAAAG-3′; sh-3: 5′-GCAAAGGCCTACTTCCCATGG-3′.

### 2.7 Quantitative Real-Time PCR (qRT-PCR)

Total cellular RNA was extracted using TRIzol™ reagent (Invitrogen, Thermo Fisher Scientific, 15596026CN, Waltham, MA, USA) according to the manufacturer’s instructions. For reverse transcription, 1 μg of total RNA was used as input. cDNA was reverse transcribed from RNA using the SuperScript™ III First-Strand Synthesis System (Thermo Fisher Scientific, 18080051, Waltham, MA, USA). The reverse transcription reaction was performed in a total volume of 20 μL, with conditions as follows: 25°C for 5 min, 50°C for 60 min, and 70°C for 15 min. qRT-PCR assays were subsequently performed using PowerUp SYBR Green Master Mix (Thermo Fisher Scientific, A25742, Waltham, MA, USA) for qRT-PCR detection according to the manufacturer’s protocol. All qRT-PCR reactions were carried out on a QuantStudio 3 Real-Time PCR System (Thermo Fisher Scientific, Waltham, MA, USA). The thermal cycling conditions were: initial denaturation at 95°C for 2 min, followed by 40 cycles of 95°C for 15 s (denaturation), 60°C for 30 s (annealing), and 72°C for 30 s (extension). A melting curve analysis was performed to verify product specificity. The primer sequences used in this study are listed in Table 1. The 2^−ΔΔCt^ method was used to quantify the relevant mRNA expression of target genes, with GAPDH serving as the internal reference gene. All qRT-PCR reactions were performed in triplicate for each sample (technical replicates), and three independent biological replicates were analyzed.

**Table 1:** Relevant primer sequences.

Gene	Primer Sequence
POLQ	F: 5′-GTGAAGACCCGTTTACCATAGA-3′,
	R: 5′-AGATCCTGTGACAATATGCTCC-3′;
E-Cadherin	F: 5′-GATTCTGCTGCTCTTGCTGTTTCTTC-3′,
	R: 5′-GGTCCTCTTCTCCGCCTCCTTC-3′;
N-cadherin	F: 5′-CTTGTGCTGATGTTTGTGGTATGGATG-3′,
	R: 5′-AGTCATAGTCCTGGTCTTCTTCTCCTC-3′;
Vimentin	F: 5′-TCGTGAATACCAAGACCTGCTCAATG-3′,
	R: 5′-ACAACTGGAATGCTCTGGATGTAACTC-3′;
c-Myc	F: 5′-CTGAGGAGGAACAAGAAGATGAGGAAG-3′,
	R: 5′-TCCAGCAGAAGGTGATCCAGACTC-3′;
Cyclin D1	F: 5′-GCCCTCGGTGTCCTACTTCAAATG-3′,
	R: 5′-TCCTCCTCGCACTTCTGTTCCTC-3′;
GAPDH	F: 5′-CTCACCGGATGCACCAATGTT-3′,
	R: 5′-CGCGTTGCTCACAATGTTCAT-3′;

### 2.8 Western Blot Analysis

Cells were washed twice with PBS, followed by cell lysis using RIPA buffer (Beyotime; Biotechnology, P0013B, Shanghai, China) containing the protease inhibitor cocktail (Yeasen; Biotechnology, 20101ES03, Shanghai, China) to extract total protein. Protein concentration was determined using a BCA assay kit (Beyotime; Biotechnology, P0012, Shanghai, China). Equal amounts of protein (30 μg per lane) were loaded and separated by 10% SDS-PAGE gel electrophoresis using a Mini-PROTEAN Tetra Vertical Electrophoresis Cell (Bio-Rad Laboratories, 1658004, Hercules, CA, USA). Proteins were then transferred from the gel to polyvinylidene fluoride (PVDF) membranes (Merck Millipore, IPVH00010, Burlington, MA, USA) using a Trans-Blot Turbo Transfer System (Bio-Rad Laboratories, 1704150, Hercules, CA, USA). After being blocked with 5% skimmed milk powder (BD Biosciences, 232100, Franklin Lakes, NJ, USA) in TBST (Tris-buffered saline with Tween 20; Biosharp, BL602A, Hefei, China) for 1 h at room temperature, the membranes were incubated with the corresponding primary antibodies at 4°C overnight: anti-POLQ (Proteintech, 28590-1-AP, 1:800, Rosemont, IL, USA), anti-β-catenin (Cell Signaling Technology, 8480S, 1:1000, Danvers, MA, USA), anti-c-Myc (Proteintech, 10828-1-AP, 1:1000, Wuhan, China), anti-Cyclin D1 (Proteintech, 60186-1-Ig, 1:1000, Wuhan, China), anti-APC (Santa Cruz Biotechnology, sc-9998, 1:500, Dallas, TX, USA), anti-GSK-3β (Cell Signaling Technology, 12456S, 1:1000, Danvers, MA, USA), anti-E-cadherin (Proteintech, 20874-1-AP, 1:1000, Wuhan, China), anti-N-cadherin (Proteintech, 22018-1-AP, 1:1000, Wuhan, China), anti-Vimentin (Proteintech, 10366-1-AP, 1:1000, Wuhan, China), and anti-GAPDH (Proteintech, 60004-1-Ig, 1:5000, Wuhan, China). The membranes were washed three times with Tris buffered saline containing Tween20 (TBST), followed by incubation with horseradish peroxidase (HRP)-labeled secondary antibodies (Proteintech, SA00001-1 (anti-rabbit) and SA00001-2 (anti-mouse), 1:5000, Wuhan, China) for 1 h at room temperature. Finally, the target protein bands were detected using ECL chemiluminescent reagent (Beyotime; Biotechnology, P0018FS, Shanghai, China) Chemiluminescent signals were captured using a ChemiDoc MP Imaging System (Bio-Rad Laboratories, 12003154, Hercules, CA, USA). Each sample was loaded in duplicate as technical replicates, and band intensities were quantified as gray values using ImageJ software (National Institutes of Health, version 1.53c, Bethesda, MD, USA). Each experiment was independently repeated three times (three biological replicates), with representative blots shown.

### 2.9 Clinical Tissue Samples

A total of 55 paired colorectal cancer (CRC) and adjacent normal tissue samples were obtained from patients who underwent surgical resection at the Fifth Affiliated Hospital of Anhui Medical University (Fuyang, China) between January 2020 and December 2022. All specimens were histopathologically confirmed by two independent pathologists. Adjacent normal tissues were taken at least 3–5 cm away from the tumor margin. Fresh tissues were immediately snap-frozen in liquid nitrogen and stored at −80°C until RNA extraction. Informed consent was obtained from all patients, and the study protocol was approved by the Ethics Committee of Anhui Medical University (approval No. KY2023014). This study was conducted in full compliance with the Declaration of Helsinki and all applicable national and institutional guidelines.

### 2.10 Immunohistochemistry

All human tissue samples were obtained from 55 patients with colorectal cancer who underwent surgical resection at The Fifth Affiliated Hospital of Anhui Medical University between January 2020 and December 2022. Inclusion criteria were: pathologically confirmed primary colorectal adenocarcinoma, no preoperative radiotherapy or chemotherapy, and availability of complete clinical and follow-up data. The study was approved by the Ethics Committee of Anhui Medical University (Approval Number: KY2023014), and written informed consent was obtained from all participants. Fresh tissue blocks (≤2 cm × 1.5 cm × 0.3 cm) were collected and fixed at room temperature for 24 h in 10 times their volume of 10% neutral buffered formalin. Routine dehydration, clearing, and paraffin embedding were performed. Sections of 4 μm thickness were prepared and baked at 60°C for 2 h. The sections were dehydrated with xylene, rehydrated using a graded alcohol series, and washed with PBS. Perform high-pressure antigen repair with sodium citrate buffer (pH 6.0) for 2 min, block endogenous peroxidase with 3% hydrogen peroxide at room temperature for 30 min. The sections were then incubated with primary antibody against POLQ (Proteintech, 28590-1-AP, 1:800, Rosemont, IL, USA) at 4°C overnight. After washing, reaction enhancer (Beyotime Biotechnology, P0260, Shanghai, China) and HRP-labeled goat anti-rabbit IgG (H + L) (Beyotime Biotechnology, A0208, Shanghai, China) were added successively. Staining was developed with DAB chromogen (Beyotime Biotechnology, P0202, Shanghai, China) for 4 min, counterstained with hematoxylin (Beyotime Biotechnology, C0107, Shanghai, China), and mounted. Two pathologists score it in a double-blind manner: multiply the staining intensity (0–3 points) by the positive cell area (0–3 points), with a total score of 0–2 points indicating low expression and 3–9 points indicating high expression. SPSS software was used to analyze the relationship between clinical characteristics and POLQ expression: categorical variables were analyzed using the chi-square test or Fisher’s exact test, while continuous variables were first subjected to a normality test, followed by the *t*-test for normally distributed data or the Mann-Whitney U test for non-normally distributed data. All reported *p*-values were two-sided, and *p* < 0.05 was considered statistically significant (no adjustment for multiple comparisons was applied).

### 2.11 Immunofluorescence

SW480 and HCT116 cells (transduced with POLQ overexpression vector or control vector as described in Cell transfection) were seeded on glass coverslips in 24-well plates and cultured for 48 h. Cells were then washed twice with PBS, fixed with 4% paraformaldehyde (Beyotime Biotechnology, P0099, Shanghai, China) for 15 min at room temperature, and permeabilized with 0.1% Triton X-100 (Beyotime Biotechnology, P0096, Shanghai, China) in PBS for 10 min. After slightly drying the treated cell slides, draw circles with a staining pen. Add 100 μL of Immunostaining Permeabilization Buffer with Triton X-100 (Beyotime Biotechnology, P0096) to the circled areas and incubate for 20 min at room temperature. Wash with PBS three times. Then, add blocking solution (3% bovine serum albumin (BSA; Beyotime Biotechnology, ST023, Shanghai, China) in PBS) at room temperature for 30 min. For primary antibody of goat origin, 10% donkey serum was used; otherwise, 3% BSA was used. Next, add the primary antibody against β-catenin (Cell Signaling Technology, 8480S, 1:200 dilution, Danvers, MA, USA) and incubate at 4°C in a humid box overnight. Wash with PBS three times, then add the corresponding species’ fluorescent secondary antibody (Alexa Fluor 488-conjugated anti-rabbit IgG; Thermo Fisher Scientific, A32731, 1:500 dilution, Waltham, MA, USA) and incubate at room temperature in the dark for 50 min. Wash with PBS three times again, add DAPI staining solution (Beyotime Biotechnology, C1005, 1 μg/mL final concentration, Shanghai, China), and stain the nuclei in the dark for 10 min. Finally, the slides were washed three times with PBS, slightly dried, and mounted using an anti-fade mounting medium (Beyotime Biotechnology, P0126, Shanghai, China). Fluorescence images were captured using a Zeiss LSM 800 confocal microscope (Carl Zeiss, Jena, Germany) with a 63× oil immersion objective. For each experimental condition, at least five random microscopic fields per coverslip were captured and analyzed as technical replicates. Each experiment was independently repeated three times (three biological replicates), and representative images are shown.

### 2.12 Dual Luciferase Reporter Assay

To evaluate the effect of POLQ on the transcriptional activity of the Wnt signaling pathway, we performed a dual luciferase reporter assay using TOPflash (TCF/LEF binding sites) and FOPflash (mutant negative control) reporter plasmids (Addgene, TOPflash: 12456, FOPflash: 12457, Watertown, MA, USA). The pRL-TK Renilla luciferase internal control plasmid was purchased from Promega Corporation (E2241, Madison, WI, USA). Stable POLQ-knockdown (shRNA) and stable POLQ-overexpressing SW480 and HCT116 cell lines (constructed via lentiviral transduction as described in Cell transfection) were used. The SW480 and HCT116 cells were seeded in 24-well plates (1 × 10^5^ cells per well), and cultured overnight. Then, using Lipofectamine 3000 transfection reagent (Thermo Fisher Scientific, L3000015, Waltham, MA, USA), the following plasmids were co-transfected in different groups: 200 ng TOPflash or FOPflash, 20 ng pRL-TK internal control plasmid. No additional POLQ expression or knockdown vectors were co-transfected, as POLQ modification had been stably established. After 48 h, cells were lysed, and dual luciferase activities were measured using the Dual-Lumi™ Dual Luciferase Reporter Gene Assay Kit (Beyotime, RG088S, Shanghai, China). Briefly, 20 μL of cell lysate was mixed with 100 μL of firefly luciferase detection reagent, incubated for 10 min, and luminescence intensity was measured using a GloMax 20/20 Luminometer (Promega, E5311, Madison, WI, USA); then 100 μL of Renilla luciferase detection reagent was added, incubated for another 10 min, and luminescence was measured again using the same luminometer. The ratio of firefly luciferase activity to Renilla luciferase activity (Luc/Rluc) was calculated. Three technical replicates were set for each group, and the experiment was independently repeated three times (three biological replicates).

### 2.13 Response to the Experiment

For rescue experiments, stable POLQ-overexpressing and stable POLQ-knockdown SW480 and HCT116 cell lines (established via lentiviral transduction as described in Cell transfection) were used. The cells were divided into the following groups: (1) DMSO control, (2) stable POLQ-overexpressing, and (3) stable POLQ-overexpressing combined with Wnt inhibitor XAV939. For POLQ knockdown combined with Wnt activation experiment, the cells were divided into: (1) DMSO control, (2) stable POLQ-knockdown (shRNA), and (3) stable POLQ-knockdown combined with Wnt activator SKL2001. No transient transfection of pcDNA3.1-POLQ or siRNA was performed, as POLQ modification had been stably established. XAV939 (Selleck Chemicals, S1180, Houston, TX, USA) and SKL2001 (Selleck Chemicals, S7220, Houston, TX, USA) were dissolved in dimethyl sulfoxide (DMSO; Sigma-Aldrich, D2650, St. Louis, MO, USA) to prepare 10 mm and 50 mm stock solutions, respectively, and stored at −20°C. For experiments, XAV939 was used at a final concentration of 2 μm, and SKL2001 at 20 μm. XAV939 treatment was initiated and continued for 48 h; SKL2001 treatment was initiated and continued for 48 h. The DMSO control group received an equivalent volume of DMSO vehicle (final DMSO concentration ≤ 0.1%). Transwell chambers with 8.0 μm pore size polycarbonate membranes (Corning Incorporated, 3422, Corning, NY, USA) were used for both migration and invasion assays. After incubation, non-migrating or non-invading cells on the upper surface of the membrane were gently removed with a cotton swab. Cells (4 × 10^4^/well) were seeded in the upper chamber. For invasion assays, the chamber was pre-coated with Matrigel (Corning, 356234) diluted 1:8 (50 μL/chamber, 37°C for 1 h). After 24 h (migration) or 48 h (invasion), cells on the membrane were fixed with 4% paraformaldehyde at room temperature for 20 min and stained with 0.1% crystal violet at room temperature for 15 min. Five random fields (200×) per membrane were quantified using ImageJ (NIH, v1.53c, Bethesda, MD, USA). Cells (5 × 10^3^/well) were seeded in 96-well plates. CCK-8 reagent (Beyotime, Biotechnology, C0037, Shanghai, China) was added at 0, 24, 48, and 72 h, followed by incubation at 37°C for 1 h. A 1-h incubation time was determined based on preliminary experiments showing linear absorbance readings. Absorbance at 450 nm was measured using a microplate reader (Beyotime Biotechnology, HBS-1096B, Shanghai, China). Each experiment was independently repeated three times.

### 2.14 Cell Viability Assay

Stable POLQ-overexpressing or stable POLQ-knockdown SW480 and HCT116 cells (constructed as described in Cell transfection) were seeded in 96-well plates at a density of 5 × 10^3^/well. Cell viability was assayed using the Cell Counting Kit-8 (CCK-8) kit (Beyotime; Biotechnology, C0037, Shanghai, China). Cells were cultured in RPMI-1640 medium containing 10% FBS and 1% P/S at 37°C in a 5% CO_2_ humidified incubator (as described in Cell lines and cell culture). For each experimental condition, three replicate wells were used per time point. At 0, 24, 48, and 72 h, 10 μL of CCK-8 reagent was added to each well, followed by an additional 1 h incubation. For background correction, wells containing only culture medium without cells (blank wells) were used, and their absorbance values were subtracted from all experimental wells. After the incubation, the absorbance was measured at 450 nm on a microplate reader (Beyotime Biotechnology, HBS-1096B, Shanghai, China) to assess the cell viability. Each experiment was repeated independently at least three times.

### 2.15 Transwell Assay

For transwell assay, Transwell inserts (24-well plate format, 8.0 μm pore size, Corning, NY, USA) were used. CRC cells were resuspended in serum-free medium and then inoculated in the upper chamber of a 24-well plate at 4 × 10^4^ cells per well. The lower chamber was supplemented with 600 μL of medium containing 20% FBS. After incubation at 37°C for 24 h (migration assay) or 48 h (invasion assay), non-migrating or non-invading cells on the upper surface of the membrane were gently removed with a cotton swab. Then cells migrating to the membrane of the lower chamber were fixed with 4% paraformaldehyde at room temperature for 20 min and stained with 0.1% crystal violet. For the invasion assay, the upper chamber was pre-coated with Matrigel diluted 1:8 in serum-free medium (Corning, 356234, NY, USA); 50 μL of this mixture was applied to each insert, and the plates were incubated at 37°C for 1 h to allow the gel to solidify. For the migration experiment, the chamber without the applied matrix gel is used. After the migration experiment (incubation for 24 h) or the invasion experiment (incubation for 48 h), proceed as follows: Fix, stain, and count the cells on the surface of the lower chamber membrane. The chambers were observed under a microscope and photographed (CKX53, Olympus, Tokyo, Japan), and the migrating cells were counted using ImageJ software (National Institutes of Health, version 1.53c, Bethesda, MD, USA). Each experimental condition was performed in triplicate wells.

### 2.16 Colony Formation Assay

Stable POLQ-overexpressing or stable POLQ-knockdown SW480 and HCT116 cells (constructed as described in Cell transfection) were seeded in 96-well plates at a density of 5 × 10^3^/well. The transfected cells in logarithmic growth phase and the corresponding control cell suspension were inoculated in 6-well plates at a density of 500 cells/well. Cells were cultured in RPMI-1640 medium (Gibco, Thermo Fisher, USA) supplemented with 10% fetal bovine serum (FBS, Gibco, USA) at 37°C in a humidified atmosphere containing 5% CO_2_. After 14 days of continuous incubation, the colonies were fixed with 4% paraformaldehyde at room temperature for 20 min and then stained with 0.1% crystal violet at room temperature for 15 min. Finally, colonies were imaged using a digital camera (EOS 600D, Canon, Tokyo, Japan) and quantified using ImageJ software (National Institutes of Health, version 1.53c, Bethesda, MD, USA). A colony was defined as a cluster containing at least 50 cells with clear boundaries under microscopic examination. Each experimental condition was performed in triplicate wells.

### 2.17 Statistical Analysis

All experiments were performed in at least three independent replicates, and data are expressed as mean ± standard deviation (SD). Statistical analyses were performed using both SPSS 20.0 software (SPSS Inc., Chicago, IL, USA) and R (v4.2.0, R Foundation for Statistical Computing, Vienna, Austria), specific single-cell analyses were performed using the Seurat package (v4.1.1; Satija Lab, New York, NY, USA) within the R environment. Comparisons between different groups were conducted using Student’s *t*-test, one-way analysis of variance (ANOVA), or the Kruskal–Wallis/Wilcoxon rank-sum test as appropriate; where applicable, *p*-values were adjusted for multiple comparisons using the Benjamini–Hochberg procedure. Associations between variables were assessed using Spearman correlation coefficients. Survival curves were generated using the Kaplan–Meier method. A two-sided *p*-value (or *p*-value for standard comparisons) < 0.05 was considered statistically significant. For analyses requiring dichotomization of POLQ expression (e.g., survival analysis and clinicopathological correlation), the median expression level was used as the cutoff to define high- and low-expression groups.

## 3 Results

### 3.1 POLQ Is Highly Expressed in Colorectal Cancer and Associated with Poor Prognosis in Advanced-Stage Patients

**Figure 1:**
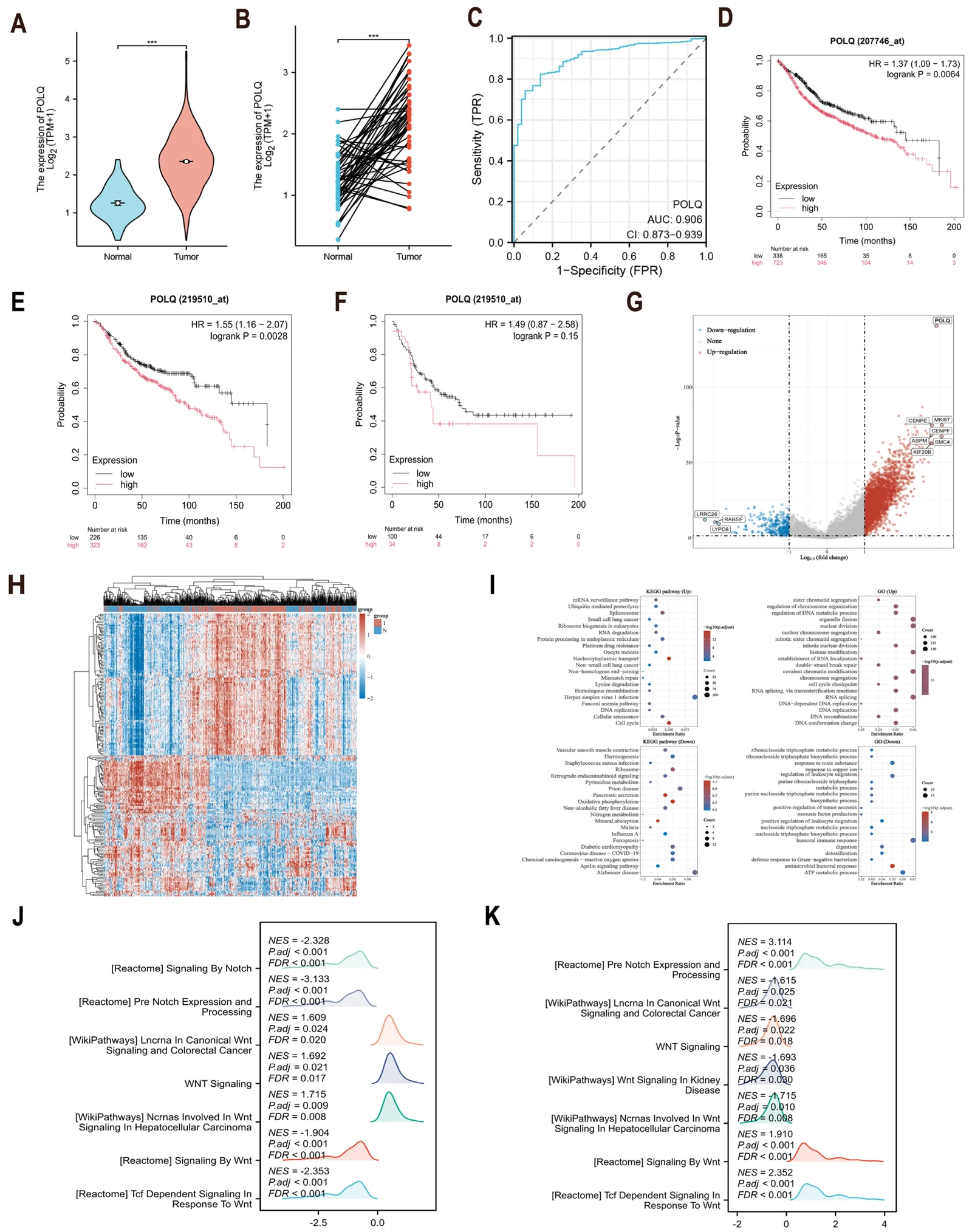
Expression characteristics, prognostic value, and pathway enrichment analysis of POLQ in colorectal cancer. (**A**,**B**) Comparison of POLQ expression between normal and tumor tissues. (**C**) ROC curve of POLQ for diagnosing CRC (AUC = 0.906). (**D**) Kaplan-Meier survival analysis indicating that elevated POLQ levels were significantly associated with poorer patient prognosis (*p* = 0.0064). (**E**) The stratified analysis revealed that the predictive value of POLQ was particularly evident in T3 cases. (**F**) No significant association was observed in T4 patients. (**G**) Volcano plot illustrating differential gene expression patterns between malignant and healthy tissues. (**H**) Heatmap of transcriptomic profiles between high and low POLQ expression groups. (**I**) KEGG/GO enrichment analysis reveals enrichment of cell cycle and DNA repair pathways in samples with high POLQ expression. (**J**) GSEA identified that the Wnt signaling pathway was particularly active in samples with high POLQ expression. (**K**) GSEA revealed negative enrichment (NES < 0) of Wnt pathway gene sets in the POLQ low-expression group when the high-expression group was used as the reference, confirming the reciprocal relationship between POLQ expression and Wnt pathway activity. ***p < 0.001.

To elucidate the clinical significance of POLQ, we systematically analyzed its expression pattern and prognostic value in colorectal cancer (CRC). Our analysis revealed that POLQ expression was significantly upregulated in tumor tissues (Fig. 1A,B). The area under the curve (AUC) for diagnosing CRC versus normal tissue reached 0.906 (Fig. 1C), indicating excellent diagnostic potential for POLQ.

Survival analysis revealed that high POLQ expression was associated with shortened overall survival (OS) in patients (*p* = 0.0064). The subgroup analysis further demonstrated that the prognostic effect of POLQ was particularly evident in patients with stage T3, while no significant association was observed in the T4 subgroup (Fig. 1D–F). In conclusion, these findings indicate that POLQ is overexpressed in CRC, and its upregulation is associated with adverse clinical outcomes.

### 3.2 Functional Enrichment Analysis Suggests POLQ Promotes Tumor Progression via Cell Cycle and Wnt/EMT Pathways

To explore the biological functions of POLQ, we analyzed differentially expressed genes (DEGs). A volcano plot displayed the significant DEG profile (Fig. 1G), and a heatmap further confirmed distinct transcriptional differences between POLQ-high and POLQ-low groups (Fig. 1H).

KEGG and GO functional enrichment analyses revealed that POLQ-high samples were significantly enriched in pathways related to cell cycle and genomic stability, including “mitotic nuclear division,” “sister chromatid segregation,” “DNA replication,” and “DNA double-strand break repair” (Fig. 1I). GSEA further demonstrated that, compared with the POLQ low-expression group, the Wnt signaling pathway-related gene sets in the POLQ high-expression group were significantly enriched (|Normalized Enrichment Score (NES)| > 1, *p* < 0.05, false discovery rate (FDR) < 0.25) (Fig. 1J); Interestingly, the reverse analysis, using the POLQ high-expression group as the reference, showed that Wnt pathway gene sets in the POLQ low-expression group were negatively enriched (Fig. 1K, NES < 0). This reciprocal pattern confirms that POLQ expression levels are positively correlated with Wnt signaling pathway activity. These results collectively suggest that POLQ may promote tumorigenesis and progression of colorectal cancer through the dysregulation of cell cycle-related processes and the modulation of the Wnt signaling pathway.

### 3.3 Single-Cell Analysis Confirms Specific High Expression of POLQ in Cancer Cells

**Figure 2:**
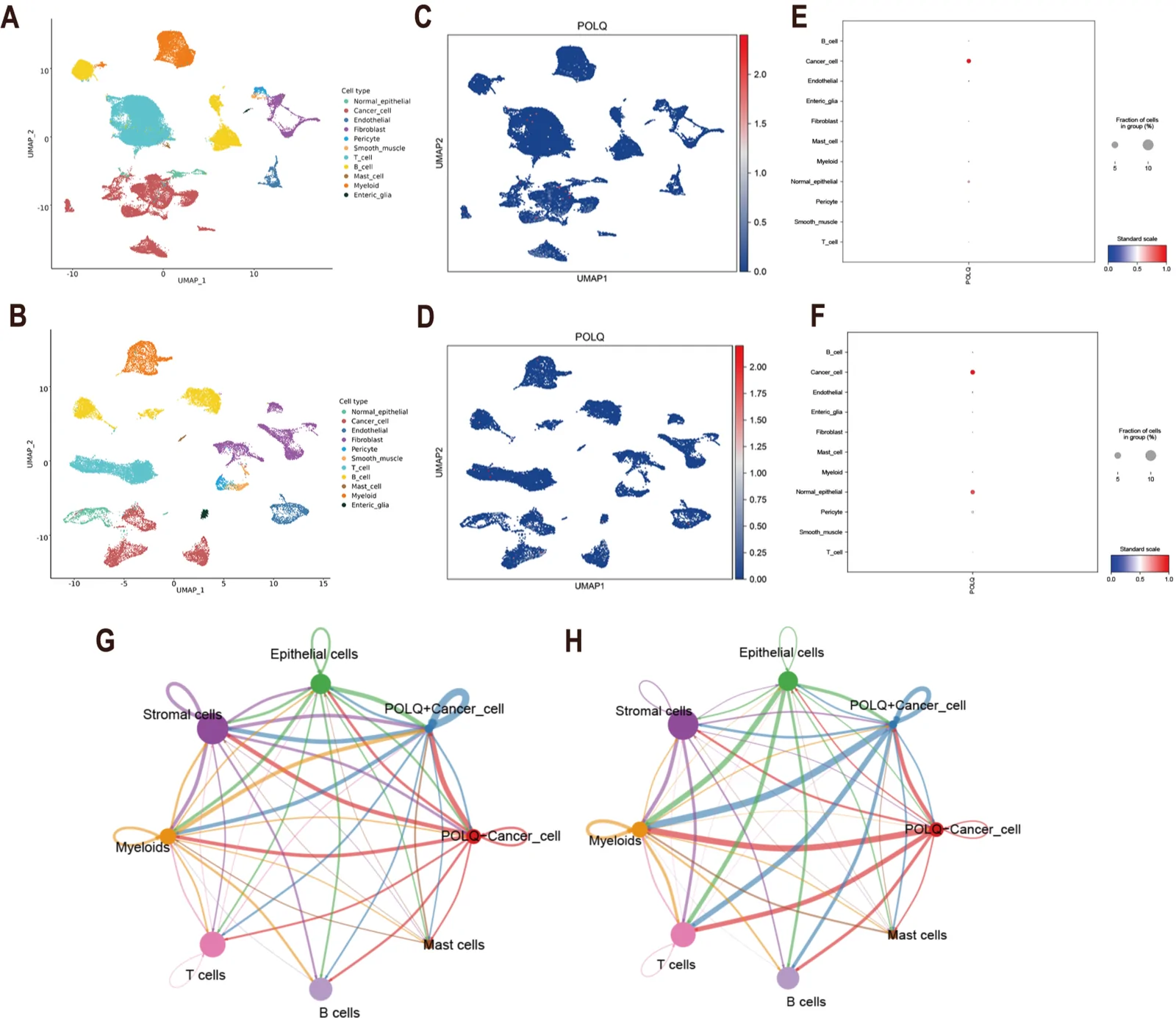
Functional enrichment and single-cell analyses reveal that POLQ regulates the cell cycle and Wnt/EMT pathways and is specifically overexpressed in cancer cells. (**A**) Clustering analysis of TME cellular composition based on an independent CRC single-cell dataset. (**B**) Distribution of major cell populations. (**C**) POLQ expression exhibits marked cell-type specificity. (**D**) POLQ expression is primarily localized to malignant epithelial cells. (**E**) The proportion of POLQ-expressing cells is significantly higher in cancer cells than in other TME cell types. (**F**) The intensity of POLQ expression in cancer cells is significantly higher than in other TME cell types. (**G**) Interaction network analysis indicates that high-POLQ-expressing cells act as key communication hubs within the TME. (**H**) These populations serve as central nodes in the TME communication network.

To characterize POLQ expression at the single-cell level, we performed an integrated analysis of two independent CRC single-cell datasets (GSE132465 and GSE144735). Unsupervised clustering resolved the major cellular constituents of the TME in both datasets, including cancer cells, normal epithelial cells, stromal cells, and various immune cells (Fig. 2A,B). Gene expression visualization revealed a striking cell-type specificity for POLQ. POLQ expression was markedly enriched in malignant epithelial cell clusters, whereas it was negligible or undetectable in normal epithelial, immune, and stromal cells (Fig. 2C,D). This pattern was further corroborated by quantitative analysis: the cancer cell population exhibited significantly higher POLQ expression compared to other cell types, regarding both the fraction of expressing cells and the mean expression intensity (Fig. 2E,F).

### 3.4 POLQ-High Cancer Cells Drive Microenvironment Remodeling via Wnt Signaling

Based on the cell-specific expression of POLQ, we further utilized CellChat to analyze the communication features of the POLQ-high cancer cell population (hereafter referred to as POLQ-high cells) within the TME.

**Figure 3:**
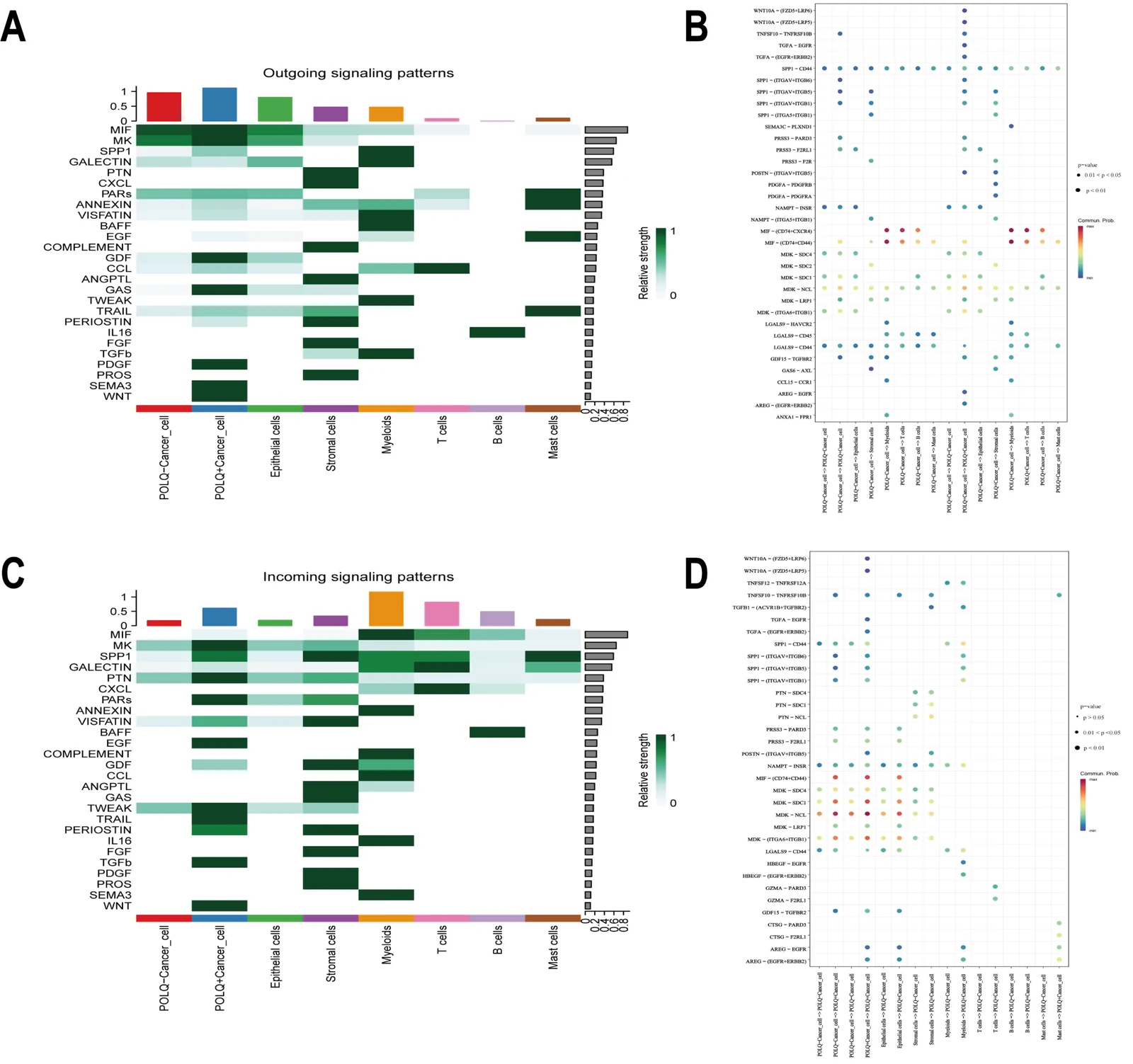
Communication Features of High POLQ-Expressing Cells and Their Inferred Regulatory Potential on the Tumor Microenvironment. (**A**) Interaction network analysis reveals that cells with high POLQ expression act as potent sources of WNT signals. (**B**) These high-POLQ-expressing cells mediate intercellular crosstalk through the WNT10A-FZD5/LRP5/6 axis. (**C**) Cells exhibiting high POLQ expression also function as signal recipients. (**D**) They display heightened sensitivity to signals from pathways, such as the TGF-β pathway, involved in EMT.

Interaction network analysis revealed that POLQ-high cells acted as communication hubs, establishing the most frequent and robust interactions with stromal cells, myeloid cells, and T cells (Fig. 2G,H). Signaling pattern analysis indicated that POLQ-high cells were exceptionally active signal senders, with the Wnt pathway being particularly prominent among their outgoing signals. Ligand-receptor analysis confirmed that this population exhibited specific high-level expression of Wnt10A and activated Wnt signaling through interaction with the FZD5 + LRP5/LRP6 receptor complex (Fig. 3A,B). Furthermore, these cells also interacted with the microenvironment by secreting other pro-tumorigenic ligands such as SPP1 and GAS6. Concurrently, POLQ-high cells also functioned as signal receivers, with their incoming signals indicating a strong receptivity to pathways like TGF-β that promote EMT (Fig. 3C,D). These findings reveal that POLQ-high cells actively remodel the TME through aberrantly activated Wnt signaling while receiving pro-EMT signals, thereby forming a communication network that drives malignant progression. Notably, these ligand-receptor interactions were statistically inferred by the CellChat algorithm using the public single-cell dataset. Further functional validation is required through experimental methods such as co-culture experiments, ligand blocking, or receptor knockout.

### 3.5 Definition of POLQ High/Low Expression and Consideration of Tumor Purity

For the TCGA-based survival and differential expression analyses, samples were stratified into high and low POLQ expression groups using the median expression value of POLQ transcripts as the cutoff. For the immunohistochemistry analysis in our cohort of 55 clinical samples, the cutoff was determined by a pathologist-scored H-score (intensity × proportion), with a total score of 0–2 classified as low expression and 3–9 as high expression, as detailed in the Methods. We recognize that POLQ is predominantly expressed in cancer cells, as evidenced by our single-cell data; consequently, its expression levels in bulk tumor samples may be influenced by tumor purity. To mitigate this, we performed an additional sensitivity analysis using the ESTIMATE algorithm on the TCGA data. We confirmed that the differential POLQ expression between tumor and normal tissues remained highly significant after adjusting for tumor purity scores (*p* < 0.001), indicating that our findings are not solely driven by differences in cellular composition.

### 3.6 Overexpression of POLQ in Colorectal Cancer Tissues and Cell Lines and Its Correlation with Aggressive Pathological Features

**Figure 4:**
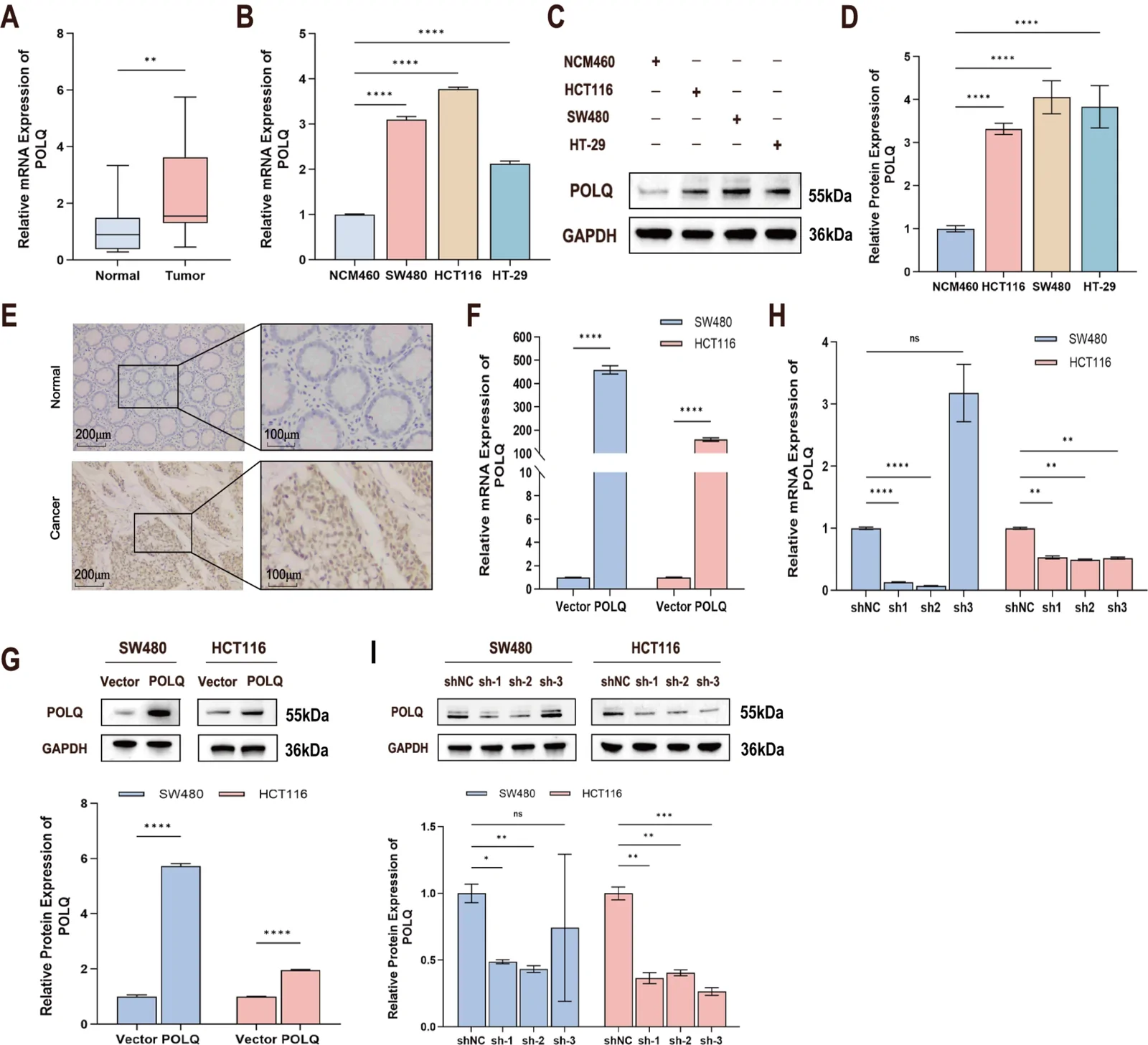
Validation of POLQ Expression in CRC Tissues and Cell Lines and Construction of Its *in vitro* Regulatory Model. (**A**) qRT-PCR analysis demonstrated significantly higher mRNA expression of POLQ in CRC tumor tissues compared to adjacent normal tissues (paired *t*-test); (**B**) The qRT-PCR analysis revealed that the POLQ mRNA level in the colorectal cancer cell lines was higher than that in the normal colon epithelial cell line NCM460. (Dunnett’s test); (**C**) The expression of POLQ protein in the above cell lines was detected using Western blotting. (**D**) Representative images show elevated POLQ protein levels in the cancer cell lines. (**E**) Expression and subcellular localization of POLQ protein were evaluated in 55 paired CRC and adjacent normal tissues via immunohistochemical staining. The definition of high vs. low expression is based on a combined intensity-proportion score (see Methods). (**F**,**G**) In SW480 and HCT116 cells, the mRNA levels in the POLQ overexpression group were significantly higher than those in the control group (unpaired *t*-test); and the protein levels in the POLQ overexpression group were also significantly upregulated (unpaired *t*-test). (**H**,**I**) In the POLQ knockdown group, the mRNA levels were significantly lower than those in the control group (unpaired *t*-test); and the protein levels were also significantly downregulated (unpaired *t*-test) (n = 3), *p < 0.05; **p < 0.01; ***p < 0.001; ****p < 0.0001, ns, not significant.

To experimentally validate our bioinformatic discovery of significant POLQ overexpression in CRC, we first examined its expression in clinical samples and cell lines. qRT-PCR analysis of 55 paired CRC and adjacent normal tissues confirmed significantly elevated POLQ mRNA levels in tumor tissues (Fig. 4A). Compared to the normal colon epithelial cell line NCM460, POLQ mRNA and protein levels were consistently higher in CRC cell lines (SW480, HCT116, HT-29) (Fig. 4B–D). These results strongly validate the bioinformatic finding of POLQ upregulation in CRC. To verify the upregulation of POLQ observed in colorectal malignancies, we conducted immunohistochemical examinations on tissue samples from 55 patients in the same study group. We found that the POLQ protein was mainly detected in colorectal cancer tissues, while its expression was extremely low or negligible in adjacent normal mucosa. Subcellular localization analysis indicated that the positive POLQ signal was mainly confined to the cell nucleus (Fig. 4E). As shown in Table 2, high POLQ expression was significantly associated with positive nerve invasion, positive vascular tumor thrombus, lymph node metastasis, T3-T4 stage, M1 stage, clinical III-IV stage, and elevated serum CEA (≥5 ng/mL) (*p* < 0.05); while there was no significant association with the patient’s gender, age, and degree of differentiation (*p* > 0.05). These results suggest that the elevated expression level of POLQ may play an important role in the tumor invasiveness, spread, and disease progression of colorectal cancer.

**Table 2:** Relationship between POLQ expression level and clinical pathological characteristics.

Variable	Total (n = 55)	POLQ Expression		χ^2^	*p* Value
		Low Expression (n = 25)	High Expression (n = 30)		
**Gender, n**				1.401	0.237
Male	29	11	18		
Female	26	14	12		
**Age, years, n**				2.645	0.104
≤60	18	11	7		
>60	37	14	23		
**Degree of differentiation, n**				0.930	0.628
Low differentiation	4	2	2		
Moderately low differentiation	7	2	5		
Moderate differentiation	44	21	23		
**Nerve invasion, n**				7.639	0.006
No	33	20	13		
Yes	22	5	17		
**Vascular carcinoma embolus, n**				7.674	0.006
No	38	22	16		
Yes	17	3	14		
**Lymph node metastasis, n**				30.556	<0.001
No	33	25	8		
Yes	22	0	22		
**T stage, n**				8.188	0.004
T1–T2	9	8	1		
T3–T4	46	17	29		
**N stage, n**				35.484	<0.001
N0	31	25	6		
N1	21	0	21		
N2	3	0	3		
**M stage, n**				4.583	0.032
Mx	50	25	25		
M1	5	0	5		
**cTNM, n**				38.194	<0.001
Stage I–Stage II	30	25	5		
Stage III–Stage IV	25	0	25		
**CEA, n**				7.185	0.007
<5 ng/mL	31	19	12		
≥5 ng/mL	24	6	18		

*p* < 0.05 indicates significance.

### 3.7 Efficiency of POLQ Overexpression and Knockdown

We assessed the mRNA and protein expression levels of POLQ overexpression and knockdown in SW480 and HCT116 cells constructed via lentiviral vectors using qRT-PCR and Western blotting. Results indicated that POLQ mRNA and protein levels were significantly higher in cells transduced with the POLQ-overexpressing lentiviral construct compared to the empty vector control group (Fig. 4F,G). In both cell lines, sh-1 and sh-2 demonstrated significantly higher knockdown efficiency than sh-3 (Fig. 4H,I). Based on comprehensive evaluation, subsequent loss-of-function experiments were performed using shNC, sh-1, and sh-2, while gain-of-function experiments were conducted using the Vector and POLQ overexpression groups.

### 3.8 POLQ Regulates Proliferation, Migration, and Invasion of CRC Cells

**Figure 5:**
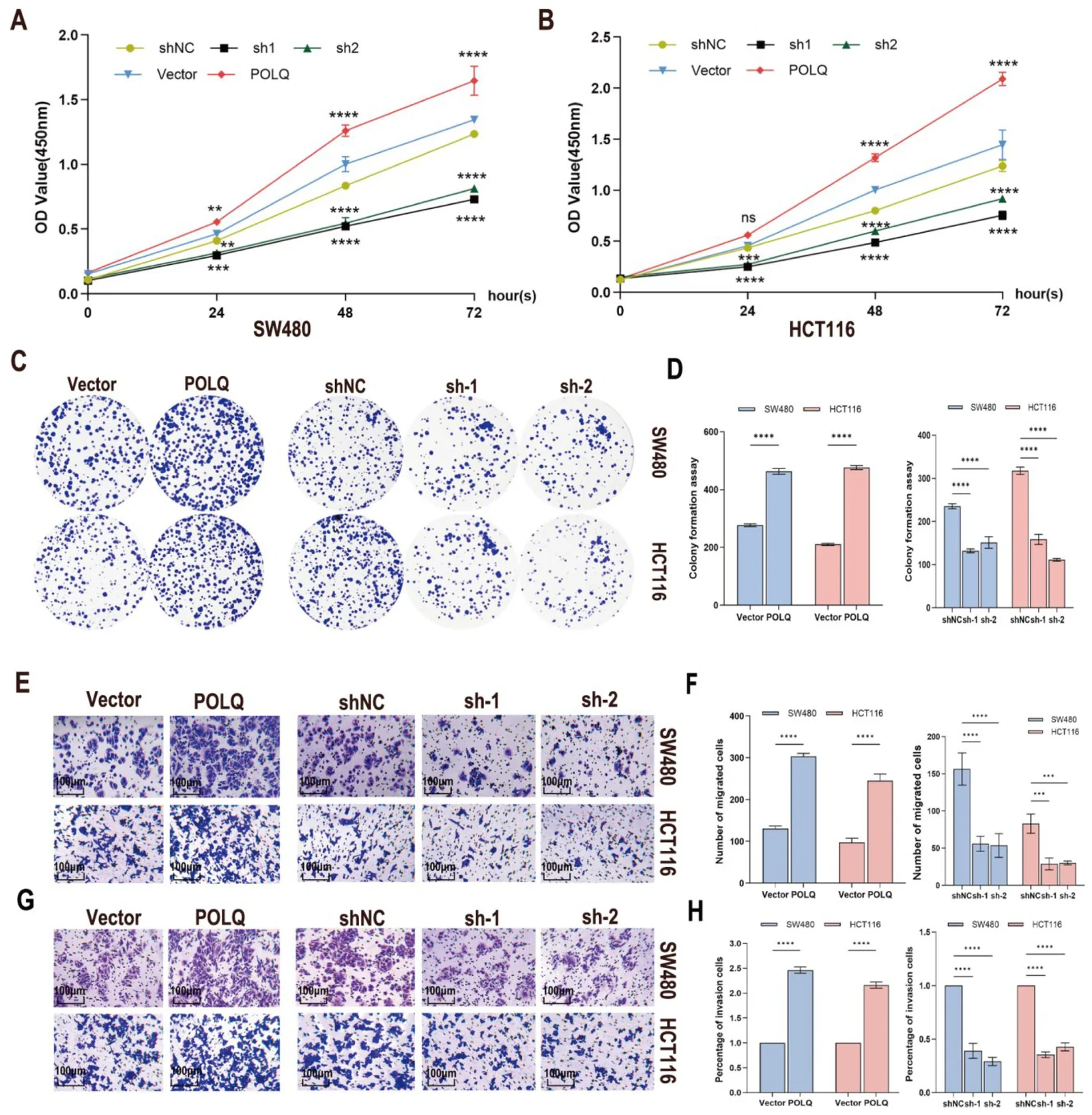
POLQ Promotes Migration, Invasion, and Proliferation of CRC Cells. (**A**,**B**) The CCK-8 experiment demonstrated that POLQ promoted the proliferation of colorectal cancer cells. Overexpression or knockdown of POLQ was performed in SW480 cells (**A**) and HCT116 cells (**B**) respectively. (Two-way ANOVA); (**C**) The colony formation experiment confirmed that POLQ can enhance the proliferation of colorectal cancer cells. (**D**) Quantitative analysis of colony numbers from three independent experiments. (Unpaired *t*-test); (**E**) The Transwell migration assay showed that overexpression of POLQ enhanced the migratory capacity of the SW480 and HCT116 cell lines, while knockdown of POLQ significantly reduced this ability; (**F**) Quantification of migrated cells from three independent Transwell assays. (Unpaired *t*-test); (**G**) The Transwell invasion assay showed that POLQ overexpression increased the invasive capacity of SW480 and HCT116 cells, while POLQ knockdown significantly reduced it; (**H**) Quantification of invading cells from three independent Transwell assays (Unpaired *t*-test), (n = 3), ns, not significant; **p < 0.01; ***p < 0.001; ****p < 0.0001.

Stable overexpression of POLQ promoted cell proliferation, while stable silencing of the POLQ gene inhibited the proliferative activity. Given the association between high POLQ expression and poor patient survival identified in our bioinformatic analysis, we hypothesized that POLQ promotes malignant phenotypes in CRC cells. CCK-8 and colony formation assays demonstrated that POLQ overexpression promoted cell proliferation, whereas POLQ silencing inhibited proliferative activity (Fig. 5A–D). Furthermore, Transwell assays revealed that POLQ overexpression significantly enhanced cell migration and invasion, whereas POLQ knockdown significantly impaired these capabilities (Fig. 5E–H). These gain- and loss-of-function experiments demonstrate that POLQ drives the proliferation, migration, and invasion of CRC cells, providing a mechanistic explanation for its association with aggressive tumor progression.

### 3.9 POLQ Regulates EMT in CRC Cell Lines

**Figure 6:**
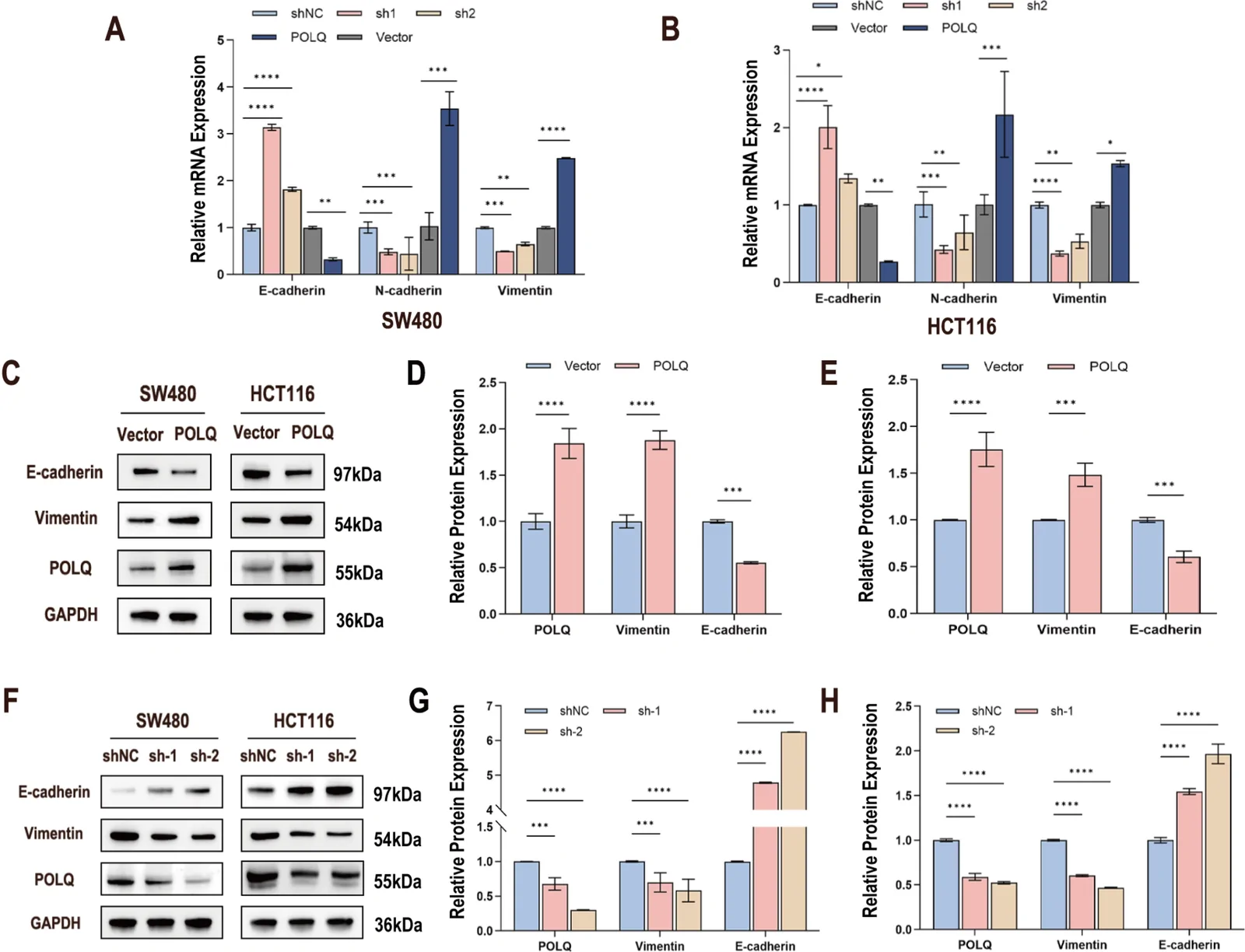
POLQ Promotes Malignant Phenotypes in CRC by Regulating EMT Markers and Cell Proliferation. (**A**) Real-time quantitative PCR assessed the impact of POLQ overexpression or knockdown on the mRNA levels of EMT markers in SW480 cells. (Unpaired *t*-test); (**B**) Real-time quantitative PCR was used to detect the effects of overexpression or knockdown of POLQ on the mRNA levels of EMT markers in HCT116 cells. (Unpaired *t*-test); (**C**) Western blotting analyzed the expression of EMT-related proteins after overexpression of POLQ. (**D**,**E**) Quantification of EMT protein expression after overexpression of POLQ in SW480 (**D**) and HCT116 (**E**) cells. (Unpaired *t*-test); (**F**) WB was used to detect the expression of EMT-related proteins after knockdown of POLQ. (**G**,**H**) Quantitative analysis of EMT protein expression after knockdown of POLQ in SW480 (**G**) and HCT116 (**H**) cells. (Unpaired *t*-test); (n = 3). *p < 0.05; **p < 0.01; ***p < 0.001; ****p < 0.0001.

Our bioinformatic analysis suggested a potential link between POLQ and the EMT. To validate this, we examined the expression of key EMT markers. In POLQ-overexpressing cells, the mRNA and protein levels of the mesenchymal markers N-cadherin and Vimentin were upregulated, while the epithelial marker E-cadherin was downregulated. Conversely, POLQ knockdown produced the opposite effects (Fig. 6A–H). These results demonstrate that POLQ induces EMT in CRC cells, validating our initial bioinformatic prediction.

### 3.10 Effect of POLQ Silencing or Overexpression on the Wnt/B-Catenin Signaling Pathway in CRC Cell Lines

**Figure 7:**
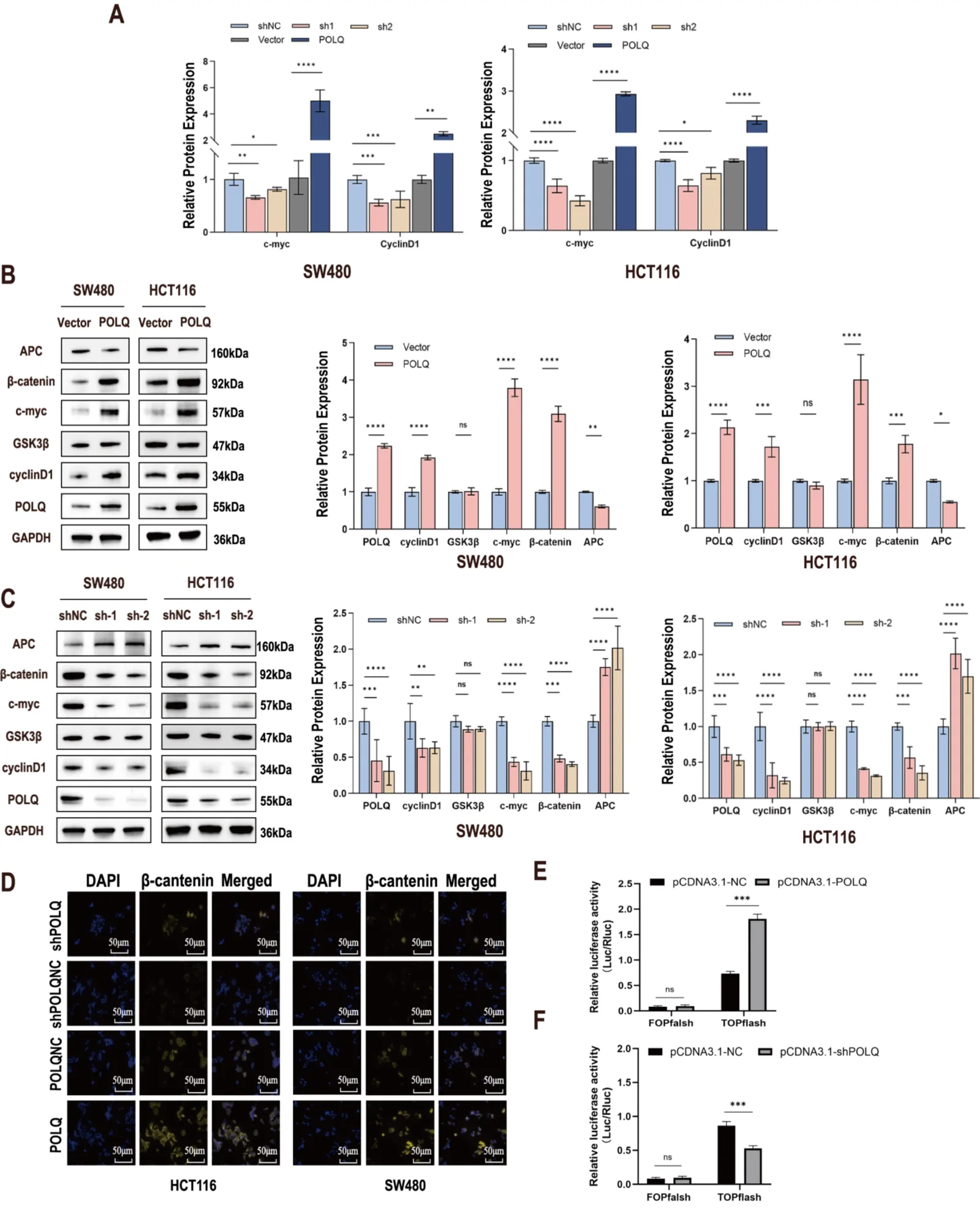
POLQ Positively Regulates the Wnt/β-Catenin Signaling Pathway and Its Downstream Target Gene Expression. (**A**) Upon POLQ overexpression, the protein levels of β-catenin, c-Myc, and Cyclin D1 all increased, while APC levels decreased; the expression of GSK-3β protein showed no significant change. (Non-paired *t*-test); (**B**) Upon POLQ knockdown, the protein levels of β-catenin, c-Myc, and Cyclin D1 all decreased, while APC levels increased; the expression of GSK-3β protein showed no significant change. (Non-paired *t*-test); (**C**) Overexpression of POLQ increased the mRNA expression levels of c-Myc and Cyclin D1, while knockdown of POLQ reduced the mRNA expression levels of c-Myc and Cyclin D1 (Unpaired *t*-test); (**D**) In SW480 and HCT116 cells, β-catenin (yellow) was diffusely distributed in the cytoplasm and cell membrane of the control group (shNC), with a relatively weak nuclear signal; in the POLQ overexpression group, the fluorescence intensity of β-catenin was significantly enhanced, and nuclear accumulation was observed. Scale bar: 50 μm. (**E**,**F**) The dual luciferase assay was used to detect the effect of POLQ on Wnt pathway activity. TOPflash (containing TCF/LEF) or mutant FOPflash was co-transfected with Renilla into cells, followed by overexpression or knockdown of POLQ (**E**) or (**F**). In the FOPflash group, there was no difference in Luc/Rluc; in the TOPflash group, overexpression of POLQ significantly increased Luc/Rluc (**E**), and knockdown significantly decreased it (**F**) (unpaired *t*-test); (n = 3), *p < 0.05; **p < 0.01; ***p < 0.001; ****p < 0.0001, ns no significance.

Our CellChat analysis predicted prominent Wnt signaling in POLQ-high cancer cells. To mechanistically investigate this prediction, we assessed the activity of the Wnt/β-catenin pathway. We found that POLQ overexpression increased the protein levels of active β-catenin and its downstream targets c-Myc and Cyclin D1, while reducing the level of APC, a component of the β-catenin destruction complex. POLQ knockdown produced the opposite effects (Fig. 7A,B). The mRNA levels of c-Myc and Cyclin D1 were also decreased upon POLQ silencing (Fig. 7C). Notably, GSK-3β expression remained unchanged. These findings provide strong evidence that POLQ activates the Wnt/β-catenin signaling pathway, experimentally validating the robust Wnt signaling predicted by our cell-cell communication network analysis.

### 3.11 POLQ Promotes the Movement of B-Catenin into the Nucleus and Enhances Its Transcriptional Function

The intracellular localization of β-catenin was evaluated by immunofluorescence. In the shNC group, β-catenin was diffusely distributed in the cytoplasm and on the cell membrane, with occasional weak nuclear signals. Overexpression of POLQ significantly enhanced the fluorescence intensity of β-catenin. Furthermore, POLQ overexpression significantly promoted the nuclear accumulation of β-catenin in both SW480 and HCT116 cell lines (Fig. 7D). Based on these data, POLQ not only increased the protein content of β-catenin but also drove it into the nucleus, thereby enhancing the downstream transcription of the Wnt/β-catenin pathway.

To further determine whether POLQ modulates the transcriptional activity of the Wnt pathway, we employed the TOPflash reporter construct and its inactive mutant, FOPflash, in dual-luciferase reporter assays. In cells transfected with FOPflash, the relative luciferase activity (Luc/Rluc) was low and comparable in all groups. In cells transfected with TOPflash, overexpression of the POLQ gene significantly increased the Luc/Rluc ratio, while compared to the control group (NC group), knockdown of the POLQ gene significantly reduced this ratio (Fig. 7E,F). These results confirm that POLQ enhances β-catenin-mediated transcriptional activity and functions as a positive regulator of downstream targets, thus reinforcing the activation of the Wnt signaling pathway.

### 3.12 POLQ Promotes the Migration, Invasion and Proliferation of CRC Cells through the Wnt Pathway

**Figure 8:**
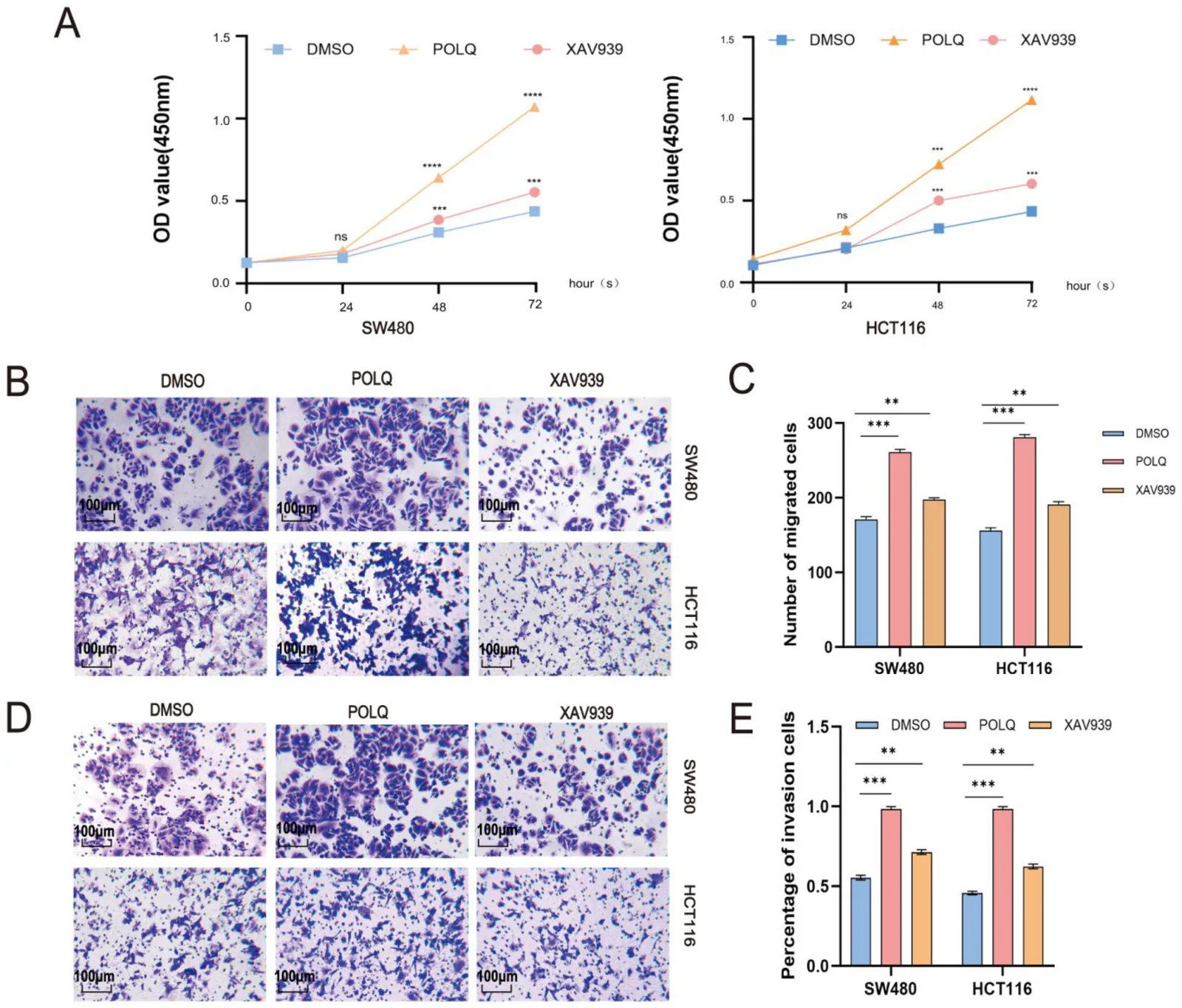
POLQ promotes the proliferation, migration and invasion of colorectal cancer cells through the Wnt/β-catenin signaling pathway. (**A**) Cell proliferation of SW480 and HCT116 cells was measured at 0, 24, 48, and 72 h in the DMSO control, POLQ overexpression, and POLQ overexpression plus Wnt pathway inhibitor (XAV939) groups. (Two-way ANOVA); (**B**) Migration of SW480 and HCT116 cell lines was assessed using Transwell chambers; (**C**) Quantitative analysis of the Transwell migration assay for SW480 and HCT116 cell lines. (one-way ANOVA); (**D**) Invasion of SW480 and HCT116 cells was evaluated via Transwell invasion assays. One-way ANOVA; (**E**) Quantitative analysis of Transwell invasion assays for SW480 and HCT116 cell lines. (n = 3), **p < 0.01; ***p < 0.001; ****p < 0.0001, ns no significance.

**Figure 9:**
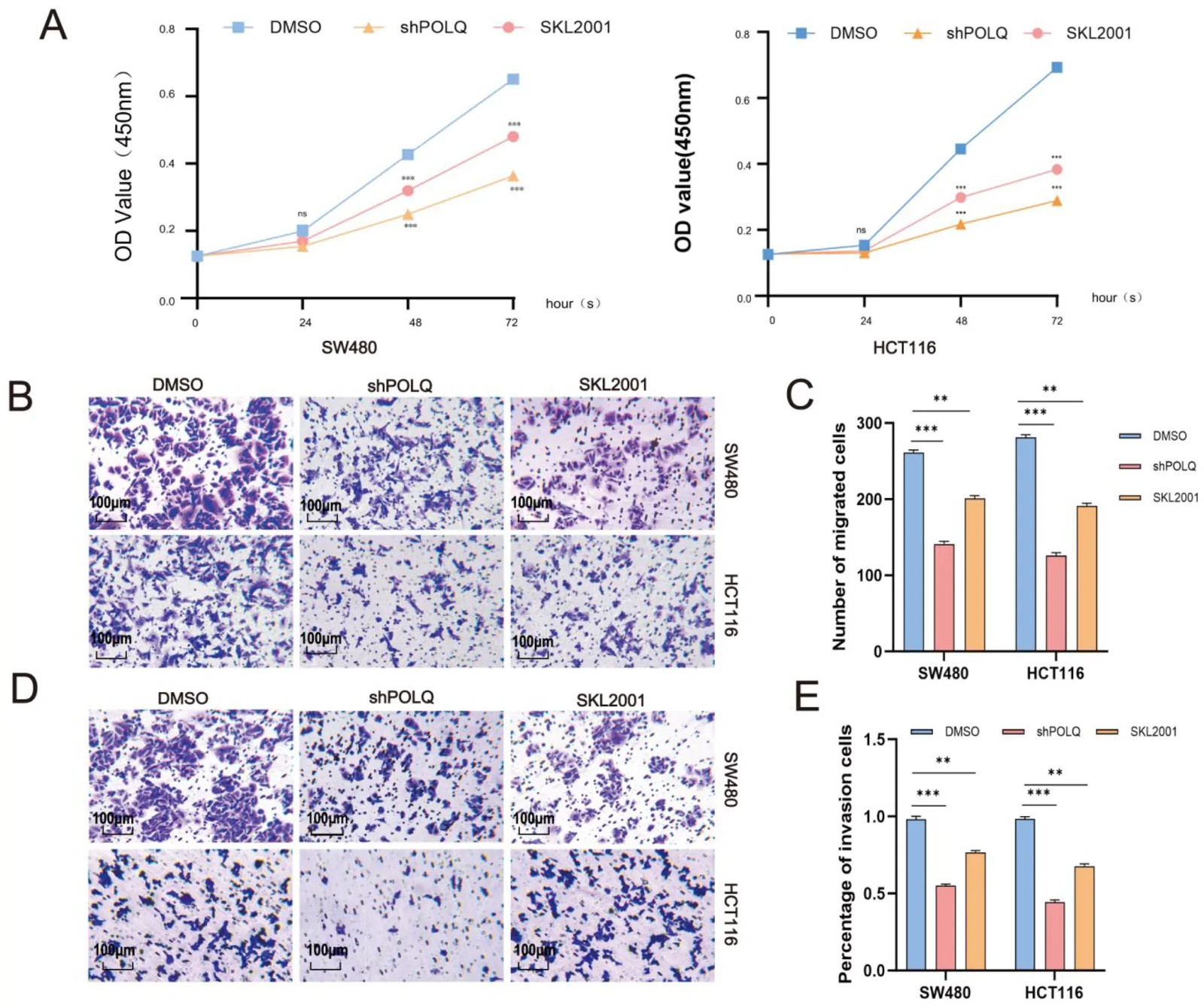
POLQ promotes the proliferation, migration and invasion of colorectal cancer cells through the Wnt/β-catenin signaling pathway. (**A**) The cell proliferation rate of the SW480 and HCT116 cell lines under three experimental conditions (DMSO control, shPOLQ knockdown, and shPOLQ knockdown plus Wnt pathway activator SKL2001 treatment) was evaluated using the CCK-8 method. Measurements were taken at 0, 24, 48, and 72 h. (two-way ANOVA); (**B**,**C**) Cell migration was assessed using Transwell assays. (One-way ANOVA); (**D**,**E**) Cell invasion was examined using Transwell assays. (One-way ANOVA); (n = 3); **p < 0.01; ***p < 0.001; ns, not significant.

To determine whether the Wnt pathway mediates the carcinogenic effect of POLQ, we conducted rescue experiments. First, we treated POLQ-overexpressing CRC cells with the Wnt inhibitor XAV939. The CCK-8 assay showed that POLQ overexpression promoted cell proliferation, as indicated by higher OD values at 24, 48 and 72 h, while XAV939 treatment significantly reversed this proliferative advantage (Fig. 8A). Based on the Transwell experiment, POLQ overexpression led to a significant increase in the number of migrating and invasive cells in the SW480 and HCT116 cell lines compared to the DMSO control group. Notably, XAV939 treatment significantly attenuated the POLQ overexpression-induced enhancement of migration and invasion (Fig. 8B–E). Conversely, we used the POLQ knockdown strategy in combination with the Wnt pathway activator SKL2001. The CCK-8 results further showed that POLQ knockdown inhibited proliferation at all three time points, and SKL2001 treatment significantly restored this phenotype (Fig. 9A). Additionally, the Transwell assay indicated that the inhibition of POLQ expression significantly reduced the cellular migratory and invasive capacities. However, SKL2001 treatment counteracted this effect, restoring the migration and invasion capacities of POLQ-deficient cells, as evidenced by the increased cell counts in Fig. 9B–E. In summary, these rescue experiments confirmed that POLQ mainly promotes the migration, invasion and proliferation of CRC cells by activating the Wnt/β-catenin signaling pathway.

## 4 Discussion

CRC ranks among the leading causes of cancer-related deaths globally, with its incidence rising among younger populations. Treatment options and efficacy for advanced-stage patients remain severely limited [1,20,21]. This study initially revealed the potential role of POLQ in CRC beyond its classical DNA damage repair function. We confirm that POLQ is significantly overexpressed in CRC and correlates with poor prognosis. Furthermore, this study has identified a new potential carcinogenic pathway: POLQ may promote the progression of CRC by activating the Wnt/β-catenin signaling pathway and inducing EMT; at the same time, single-cell network analysis suggests that it may be associated with the formation of an immunosuppressive tumor microenvironment.

In this study, POLQ protein expression was assessed using a C-terminal-targeting antibody (Proteintech, 28590-1-AP). It is important to note that full-length human POLQ comprises 2592 amino acids with a predicted molecular weight of approximately 290 kDa [23]. However, as documented in the seminal characterization of POLQ, the protein is highly susceptible to proteolysis during cell extraction, generating stable C-terminal fragments that retain the polymerase domain. Accordingly, the ~55 kDa band detected in our Western blot analyses represents the stable C-terminal proteolytic fragment of POLQ, rather than the full-length protein. This detection pattern is consistent with the biochemical properties of POLQ and with the mRNA expression changes observed in our qRT-PCR analyses (Fig. 4F–I), collectively supporting the conclusion that POLQ is transcriptionally and post-transcriptionally regulated in CRC cells. The expression of POLQ in CRC aligns with reports in other malignancies, with its potential as a prognostic biomarker being particularly pronounced in advanced patients [22,24,25]. Recent studies have identified POLQ as a novel pathogenic gene in hereditary CRC, further substantiating its pivotal role in CRC pathogenesis [26]. Our findings corroborate the known roles of POLQ in cell cycle regulation and error-prone TMEJ repair [27,28], and more importantly, reveal novel mechanisms through single-cell transcriptomics [29]. The expression of POLQ is found exclusively in malignant epithelial cells, which act as pivotal signaling hubs within the tumor microenvironment. The Wnt pathway is the most prominent outward signaling pathway in these cells. This cell-cell communication prediction prompted us to investigate whether POLQ could modulate the intracellular Wnt/β-catenin pathway activity. This study found that POLQ can upregulate the expression of β-catenin and its downstream targets c-Myc and cyclin D1, and promote the translocation of β-catenin to the nucleus and the enhancement of TCF/LEF transcriptional activity. These results suggest that POLQ may function as a novel upstream positive regulator of the Wnt pathway in colorectal cancer. Given the well-established central role of Wnt/β-catenin signaling in colorectal carcinogenesis and progression [30], the identification of POLQ as a new activator of this pathway provides a mechanistic basis for its oncogenic functions. Whether a reciprocal regulatory loop exists between POLQ and its downstream effector Cyclin D1, as suggested by recent observations in other cancer types [31], remains an open question that warrants further investigation. Mechanistic investigations provide a plausible explanation for POLQ-induced EMT through activation of the Wnt/β-catenin pathway. The modulation of EMT markers by POLQ has been demonstrated to directly enhance cellular migration and invasion capabilities [32] This finding is in contrast with the reports of ZEB1 suppressing POLQ in breast cancer [33]. Such divergent regulatory relationships may reflect context-dependent functions of POLQ across different cancer types, warranting further investigation in future studies.

This study indicates that high expression of POLQ is significantly associated with neural invasion, vascular tumor thrombus, lymph node metastasis, advanced TNM stage, and elevated serum CEA. Kaplan-Meier survival analysis shows that patients with high POLQ expression have a significantly shorter overall survival period (*p* = 0.0064). Subgroup analysis by T stage revealed that high POLQ expression was significantly associated with poor prognosis in T3 stage patients (*p* < 0.05), but not in T4 stage patients (*p* > 0.05). It is noteworthy that the sample size of T4 stage patients in the TCGA-COADREAD cohort was small and the number of death events was low, which might be due to insufficient statistical power and thus mask the true differences. Therefore, the prognostic distinction in the T3 stage subgroup was more obvious, but the negative results in the T4 stage could not rule out the potential prognostic value of POLQ. These results support the value of POLQ as a potential prognostic marker for CRC.

The significant clinical relevance of this study lies in providing novel theoretical foundations and application directions for emerging POLQ inhibitor therapies [10]. POLQ has demonstrated a “synthetic lethality” effect in HR-deficient cancers [7,34]. For instance, the POLQ inhibitor Novobiocin has been shown to effectively suppress POLQ-mediated TMEJ activity in hereditary CRC models, offering a potential therapeutic option for POLQ-mutant CRC patients [26]. Notably, recent studies have demonstrated that the FDA-approved antibiotic Novobiocin effectively suppresses POLQ-mediated TMEJ activity in hereditary CRC models, offering a potential therapeutic option for POLQ-mutant CRC patients [35]. This strategy can be extended to combined inhibition of the NHEJ and MMEJ pathways: Inhibition of DNA-PK prompts tumor cells to depend on POLQ-mediated MMEJ. Concurrent administration of POLQ inhibitors results in uncontrolled DNA end excision, leading to potent synthetic lethality in TP53-mutant models [36]. It is worth noting that this study suggests that the POLQ inhibitor, by directly interfering with the POLQ/Wnt/β-catenin oncogenic axis, may also have the potential to treat colorectal cancer with normal HR function. However, this speculation requires further verification. Furthermore, the POLQ-associated immunosuppressive microenvironment provides a rationale for combining POLQ inhibitors with immune checkpoint inhibitors. However, further research is necessary to determine the specific efficacy of POLQ inhibitors in CRC, identify optimal biomarkers, and elucidate potential resistance mechanisms.

It is important to emphasize that our observations regarding the immune microenvironment are derived exclusively from computational predictions based on public single-cell datasets. Specifically, CellChat analysis inferred that POLQ-high cancer cells may secrete Wnt ligands and respond to TGF-β signals, and these inferred communication patterns correlated with gene expression signatures suggestive of an immunosuppressive TME (e.g., reduced cytotoxic CD8^+^ T cell signatures, increased M2 macrophage-associated genes). Importantly, these findings are purely correlative and hypothesis-generating; we did not perform any experimental validation—such as multiplex immunofluorescence, immunohistochemical co-localization, or flow cytometry—on our own clinical specimens to confirm immune cell infiltration or the POLQ-immune axis. Therefore, we cannot conclude that POLQ directly “remodels” the immune landscape or drives immune evasion. Instead, our data provide a computational basis for future mechanistic investigations using immune-cell co-culture systems and immunocompetent *in vivo* models.

Our findings raise the intriguing possibility that POLQ’s oncogenic activity extends beyond its canonical repair function. We propose a cooperative “two-hit” model in which POLQ-mediated error-prone TMEJ generates genomic instability and heterogeneity, while constitutively active Wnt/β-catenin signaling acts as a pro-survival buffer that prevents apoptosis in genomically compromised cells. This synergy between mutagenic repair and survival signaling may be particularly relevant in CRC, where Wnt pathway mutations are nearly universal. Furthermore, the observation that EMT transcription factors can modulate POLQ expression suggests a potential feedback loop between POLQ, Wnt signaling, and EMT that warrants further investigation. Therapeutically, this framework supports the rationale for combining POLQ inhibitors with Wnt pathway inhibitors in advanced CRC, although this hypothesis requires rigorous experimental validation in future studies.

We acknowledge several methodological limitations that should inform the interpretation of our findings. First, all functional experiments were performed in a limited panel of two CRC cell lines (SW480 and HCT116). While these are well-established models of Wnt-driven CRC, the inherent genetic heterogeneity of the disease warrants validation in additional cell lines (e.g., RKO, LoVo) and, more importantly, in patient-derived organoids and *in vivo* models. Such studies will be essential to confirm POLQ’s tumor-promoting functions and to evaluate the efficacy of POLQ inhibitors in physiologically relevant contexts. Additionally, our mechanistic conclusions regarding the WNT10A-FZD5/LRP5/LRP6 signaling axis, derived from single-cell bioinformatic predictions, remain hypothetical and require experimental confirmation through co-culture systems, ligand blocking, and receptor knockout experiments.

Second, while our rescue experiments with XAV939 and SKL2001 support the functional relevance of the Wnt pathway in POLQ-mediated phenotypes, we did not perform genetic rescue by manipulating key nodal molecules such as β-catenin or TCF4, nor did we provide direct evidence of physical interaction between POLQ and Wnt pathway components (e.g., through Co-IP or ChIP). These approaches represent critical next steps to firmly establish the molecular architecture of the POLQ-Wnt axis. Furthermore, our proposed “dual-drive” model—linking POLQ’s error-prone repair activity to Wnt activation—currently lacks direct experimental support, such as measurements of mutation frequency, chromosomal aberrations, or TMEJ repair efficiency. Future studies employing γH2AX focus assays and reporter-based repair systems are needed to directly evaluate POLQ’s impact on genomic stability in CRC.

Third, our analysis of the tumor microenvironment and immune landscape relies on public single-cell datasets (GEO) and bioinformatic inference. We did not perform multiplex immunofluorescence or immunohistochemical co-localization (e.g., with CD8, CD68, or PD-L1) on our own clinical specimens to validate these predictions. Therefore, our suggestion that POLQ-high cells may contribute to an immunosuppressed microenvironment remains hypothesis-generating and awaits functional validation using immune-cell co-culture systems and *in vivo* models.

Fourth, our clinical findings are based on a single-center cohort of 55 patients and the retrospective TCGA dataset. While our data identify POLQ as a promising prognostic and diagnostic candidate, definitive clinical validation requires large-scale, prospective, multicenter studies with standardized assays. The prognostic value in specific subgroups (e.g., T4 stage) was limited by small sample sizes and should be interpreted with caution. Finally, the POLQ bands detected by Western blot in this study predominantly represent C-terminal proteolytic fragments (~55 kDa) rather than the full-length protein (~290 kDa), a phenomenon consistent with the inherent susceptibility of POLQ to proteolysis. Future studies using antibodies targeting the N-terminal or central domains, or expressing epitope-tagged full-length POLQ, will be necessary to confirm protein-level regulation. Despite these limitations, our findings establish a strong foundation for further investigation of POLQ as a key oncogenic driver and therapeutic target in CRC.

## 5 Conclusion

This study provides experimental evidence to clarify the role of POLQ as a pleiotropic potential oncogenic driver in colorectal cancer. We not only confirmed the specific overexpression of POLQ in CRC and its association with poor prognosis, but more importantly, revealed a previously unrecognized oncogenic mechanism: POLQ directly promotes tumor cell proliferation, migration, and invasion by activating the Wnt/β-catenin signaling pathway and inducing EMT. Concurrently, single-cell data analysis raises the hypothesis that POLQ-overexpressing cancer cells may be associated with an immunosuppressive microenvironment state; however, this inference is strictly correlational and requires experimental verification through immune profiling of clinical specimens and functional models.

Based on these findings, we propose an innovative “dual-drive” paradigm: POLQ simultaneously maintains genomic instability through the error-prone TMEJ pathway while single-cell network-based predictions suggest a potential role in immune evasion. This integrated perspective establishes POLQ as a key molecule possessing both prognostic biomarker potential and significant therapeutic target value. This study not only deepens our understanding of CRC pathogenesis but, more importantly, lays the theoretical foundation for precision therapeutic strategies targeting POLQ as a novel therapeutic avenue, pointing the way toward new research directions.

## Data Availability

The datasets generated during and/or analyzed during the current study are available from the corresponding author on reasonable request.
